# Human Umbilical Cord Mesenchymal Stem Cells: Current Literature and Role in Periodontal Regeneration

**DOI:** 10.3390/cells11071168

**Published:** 2022-03-30

**Authors:** Muhammad Saad Shaikh, Zara Shahzad, Esraa Abdulgader Tash, Omer Sefvan Janjua, Muhammad Ikram Khan, Muhammad Sohail Zafar

**Affiliations:** 1 Department of Oral Biology, Sindh Institute of Oral Health Sciences, Jinnah Sindh Medical University, Karachi 75510, Pakistan; drsaadtanvir@gmail.com; 2 Lahore Medical and Dental College, University of Health Sciences, Lahore 53400, Pakistan; zarashahzad2210@gmail.com; 3 Department of Oral and Clinical Basic Science, College of Dentistry, Taibah University, Al Madinah Al Munawarah 41311, Saudi Arabia; etash@taibahu.edu.sa; 4 Department of Maxillofacial Surgery, PMC Dental Institute, Faisalabad Medical University, Faisalabad 38000, Pakistan; osj1982@hotmail.com; 5 Private Dental Practitioner, Penrith, NSW 2750, Australia; drikramazad@gmail.com; 6 Department of Restorative Dentistry, College of Dentistry, Taibah University, Al Madinah Al Munawarah 41311, Saudi Arabia; 7 Department of Dental Materials, Islamic International Dental College, Riphah International University, Islamabad 44000, Pakistan

**Keywords:** umbilical cord mesenchymal stem cells, dental stem cells, non-dental stem cells, periodontal disease, periodontal regeneration

## Abstract

Periodontal disease can cause irreversible damage to tooth-supporting tissues such as the root cementum, periodontal ligament, and alveolar bone, eventually leading to tooth loss. While standard periodontal treatments are usually helpful in reducing disease progression, they cannot repair or replace lost periodontal tissue. Periodontal regeneration has been demonstrated to be beneficial in treating intraosseous and furcation defects to varied degrees. Cell-based treatment for periodontal regeneration will become more efficient and predictable as tissue engineering and progenitor cell biology advance, surpassing the limitations of present therapeutic techniques. Stem cells are undifferentiated cells with the ability to self-renew and differentiate into several cell types when stimulated. Mesenchymal stem cells (MSCs) have been tested for periodontal regeneration in vitro and in humans, with promising results. Human umbilical cord mesenchymal stem cells (UC-MSCs) possess a great regenerative and therapeutic potential. Their added benefits comprise ease of collection, endless source of stem cells, less immunorejection, and affordability. Further, their collection does not include the concerns associated with human embryonic stem cells. The purpose of this review is to address the most recent findings about periodontal regenerative mechanisms, different stem cells accessible for periodontal regeneration, and UC-MSCs and their involvement in periodontal regeneration.

## 1. Introduction

Periodontitis, a common and widespread condition, can cause irreparable deterioration of the tooth supporting structures and consequent tooth loss if left untreated [1]. When soft (periodontal ligament (PDL)) and hard (alveolar bone (AB) and root cementum (RC)) connective tissues, a complex anatomical structure of the periodontium, are destroyed, periodontal regeneration is of major therapeutic importance [2]. The fundamental objective of periodontal treatment is to repair the structure as well as function of the injured periodontium in a spatially defined microenvironment, as well as to manage the periodontal inflammation and development of periodontitis.

While traditional periodontal and/or surgical therapies are typically effective in limiting disease development, they cannot repair destroyed periodontal tissue or restore its functioning. Despite the use of different regenerative treatments, such as guided tissue regeneration therapy (GTR) [3,4], bone replacement grafts (BRGs) [5,6,7,8], multiple growth factor-based treatment [9,10], and enamel matrix derivative [11,12,13], the results are sometimes disappointing, with poor clinical predictability.

Periodontal regeneration has been shown to be effective in managing intraosseous and furcation defects with varying degrees of efficacy [14,15]. However, regenerative procedures are still subject to clinical failures or incomplete success due to a variety of limitations, including patient-specific factors (such as smoking, poor plaque control, etc.), improper access flaps and biomaterials applied [16], and poor periodontal training [17]. The desired therapeutic outcome is regeneration of PDL, RC, and AB in previously injured periodontium; however, this has not always been demonstrated [18]. To address these constraints, novel access flaps [19,20,21,22,23,24] and biological agents [25,26] have been created in recent years; nonetheless, clinical studies have shown a still debatable effectiveness, and histological data is often lacking. As a result, the quest for innovative regenerative procedures remains a difficult area of periodontal research.

With evolving tissue engineering and progenitor cell biology, cell-based treatment for periodontal regeneration will become more efficient and predictable, overcoming the limits of current therapeutic modalities [4]. Stem cells are appealing in this sector. They are undifferentiated cells with regenerative potential due to their power of differentiation and self-renewal into various types of cell when stimulated [27]. These cells can be inoculated directly or supplied to the defect site via cell carriers or biomaterial scaffolds [28,29,30]. Mesenchymal stem cells (MSCs) have been used in vitro and in people for periodontal regeneration, with encouraging results [31]. MSCs have been extracted and employed from dental and non-dental tissues [32]. Dental pulp stem cells (DPSCs) [33], human exfoliated deciduous tooth cells (SHEDs) [34], periodontal ligament stem cells (PDLSCs) [35], dental follicle stem cells (DFSCs) [36], and stem cells from apical papilla (SCAPs) [37] are examples of MSCs from dental tissues. Non-dental origin stem cells of various types have been employed in periodontal regeneration. The most studied stem cells have been bone marrow-derived mesenchymal stem cells (BMMSCs), embryonic stem cells (ESCs), adipose-derived stem cells (ADSCs), and induced pluripotent stem cells (iPSCs).

Human umbilical cord mesenchymal stem cells (UC-MSCs) are produced from umbilical cords and are a cheap and endless source of stem cells. Their collection does not necessitate the invasive method required for PDLSCs and does not include the concerns associated with human ESCs [38]. Furthermore, human UC-MSCs were found to be primordial MSCs with significant plasticity and developmental flexibility [39]. Furthermore, human UC-MSCs have shown little immunorejection in vivo and are not tumorigenic [39]. Because of these benefits, human UC-MSCs are a promising choice for periodontal regeneration treatment.

The purpose of this review is to discuss the current literature available related to regeneration mechanisms of periodontium, different types of stem cells available for periodontal regeneration, human UC-MSCs and their role in periodontal regeneration. This narrative review was reported using the Scale for the Assessment of Narrative Review Articles (SANRA) standards. Various databases (PubMed and Google Scholar) were examined to identify the most relevant material [40]. 

## 2. Etiopathophysiology of Periodontal Tissue and Its Degeneration 

### 2.1. The Dental Plaque and Calculus

The dental plaque-induced periodontal diseases (as the name implies) are primarily caused by tooth plaque microbes [41]. Indeed, the bacterial oral biofilm has been widely investigated and may contain up to 150 distinctive species in one individual, with up to 800 species found in human oral biofilm to date [42]. The dispute over whether species are predominantly virulent and can cause infection has raged for years and is still unanswered [43,44]. Known pathogens comprise Gram-negative anaerobes, spirochetes, and possibly viruses; however, it is more likely that dysbiosis (microbic biofilm discrepancy) is the pathogenic ‘unit’ rather than a specific pathogen [45]. If periodontal disease (PD) was caused by one or a few particular bacteria, a focused modification of the microbial plaque rather than complete biofilm clearance would be the recommended treatment approach [44]. 

In prospective cohort studies [46], aggressive PDs were related to colonization by particular clones of *Aggregatibacter actinomycetemcomitans*. Other species, including *Porphyromonas gingivalis*, have been linked to severe or advanced PD [47], although the microbial biofilm temporalty and its link to PD is less evident. A systematic review [48] showed that aggressive and chronic periodontitis (CP) might not be distinguished depending on individual periodontal infections, implying that the causal microbial biofilm in both conditions is comparable. Most people have had several viral infections, and viral DNA or RNA might still be identified in bodily tissues long after symptoms of illness have faded, and these quiescent viruses can revive during inflammatory outbursts [49]. As a result, establishing a cause–effect relationship between enhanced viral existence and PD is challenging, and links between PD and herpes viruses may just be epiphenomena [50]. As a result, the viral significance in the PD etiology remains debatable. However, when used in conjunction with conventional therapy, antiviral therapy decreased probing pocket depth (PPD) and periodontal inflammation [51], and is thus suggested for periodontal treatment by certain clinicians [52]. 

Dental plaque can be unmineralized or mineralized: supragingival plaque (present on the oral and tooth surfaces) is generally unmineralized; however, subgingival plaque (between the gingival margin and the tooth’s neck or root) is usually black in color and mineralized. Subgingival plaque mineralization is created by serum transudate ions, triggered by the periodontal tissue infection, whereas supragingival plaque mineralization is caused by salivary calcium and phosphate ions accumulated within the dental plaque [53].

### 2.2. Immunological Perspectives

**Figure 1:**
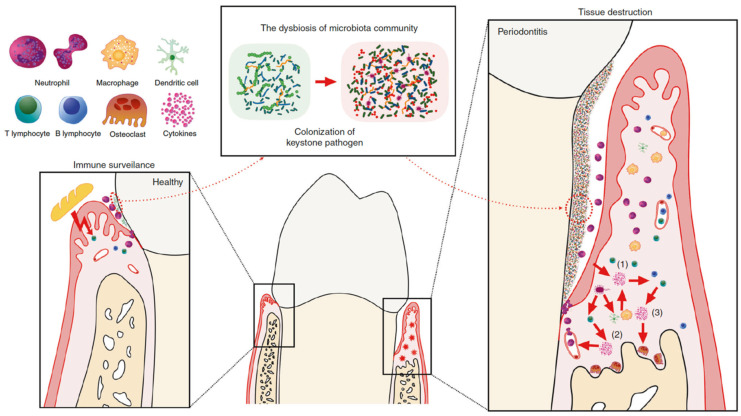
The periodontal homeostasis, CP pathophysiology, and roles of the concerned cytokines. Local challenge and a slight host immune response are balanced in health. The commensal bacteria and mechanical spur from mastication contribute to the development of local mucosal immunity. An adequate infiltrating neutrophil in the gingival sulcus, as well as several resident immune cells in the gingival tissue, such as T helper (TH) 17 cells and innate lymphoid cells, are adequate in this condition. Nonetheless, if the immunological pathogenicity of this microbiota is increased by the colonization of keystone pathogens, tissue damage occurs due to hyperactivity of the host immune response. The interface between the microbiota and host cells results in the primary wave of cytokine emission (1) that primarily contributes to the intensification of the pro-inflammatory cytokine cascade as well as the recruitment, activation, and differentiation of certain immune cells. Furthermore, mononuclear phagocytes and antigen presenting cells release a group of cytokines (2) that are strongly associated to the differentiation of a particular subset of lymphocytes after being stimulated by the microbiome. All these cell subsets secrete a unique cytokine pattern, operating as a positive-feedback factor or direct effector (3) and finally cause tissue death. The figure was adapted from Pan et al. (2019) [59].

The microbial biofilm presence may not be enough to cause PD development. Disease develops when the equilibrium between the oral bacterial biofilm and the host is disrupted (Figure 1), either due to dysbiosis or an overreaction of the host’s immune system to microbial existence [54,55,56]. This disproportion is challenging to understand because of significant differences in the microbial plaque as well as the host immunological and genetical profiles [45,57] and it culminates in aggravated inflammation causing the periodontal destruction [58].

Epithelial cells act as a physical barrier against pathological response and stimulate both the types of immunity (innate and acquired) [57]. The epithelial dendritic Langerhans cells collect microbic antigen and transport it to lymphoid tissue for presentation to lymphocytes. Infiltration of neutrophils, granulocytes, and lymphocytes into the periodontal lesion follows: neutrophils try to ingest and destroy pathogen although are overpowered by the volume and chronic perseverance of the bacterial oral biofilm. This strong chronic inflammatory reaction causes the osteoclasts to resorb AB, the matrix metalloproteinases to degrade PDL fibers, and the formation of granulation tissue [60,61]. This diseased state will not be resolved until the microbial biofilm and granulation tissue are effectively eliminated or the tooth is exfoliated.

#### T Cells: Friend or Foe?

**Figure 2:**
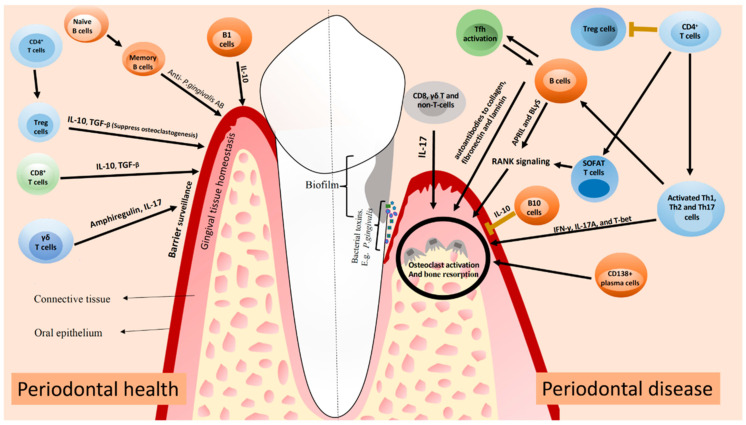
A synopsis of how the previously described T and B cells can contribute to periodontal health and disease. Treg and cytotoxic T cells (CD8+ T cells) in periodontal health contribute to periodontal homeostasis by producing IL-10 and transforming growth factor-ß (TGF-ß). To improve periodontal homeostasis, ***γδ***T cells generate amphiregulin and IL-17. B cells generate antibodies against periodontal bacteria, slowing the progression of periodontal inflammation. Activated TH1, TH2, and TH17 cells in periodontal disease release pro-inflammatory cytokines that lead to tissue destruction. T and B cells both generate receptor activator of nuclear factor ***κ*** B-Ligand (RANKL), activating osteoclasts and causing alveolar bone resorption. T-follicular helper (Tfh) cell clonal stimulation of B cells can result in the generation of autoantibodies against collagen, fibronectin, and laminin, which can contribute to local tissue damage. Periodontitis is most likely influenced by a lack of Treg cells or their dysfunction. Other cells’ production of IL-17 can also contribute to tissue injury via osteoclast activation. The figure was adapted from Figueredo et al. (2019) [65].

The antibodies (produced by B cells) are thought to be significant in periodontitis protection. T cells may contribute to cell-mediated immunity in addition to the antibody reaction by triggering multiple TH cell reactions including TH1, TH2, and TH17. TH1 cells may be involved in the early phases of CP, but TH2 cells may be crucial later on (Figure 2) [62].

Modern cytokine profiling, on the other hand, has demonstrated that several TH cells subsets including TH9, TH17, TH22, and regulatory T (Treg) cells, and cytokines (such as interleukin (IL) 17), are significant in PD immunopathology [63]. A disparity in these cell subset reactions may cause infection and may be associated with the function of leukocyte-derived EGF-like repeat and developmentally regulated endothelial cell locus 1 protein (DEL1), an endogenic inhibitor of neutrophil adherence [64], preventing IL17-induced AB loss.

### 2.3. Susceptibility of PDs

Gingivitis susceptibility may represent vulnerability to CP [66,67] and epidemiological reports show that gingivitis precedes the CP onset [68]. Additionally, the lack of gingival inflammation is an excellent predictor for long-term maintenance of periodontal health, both individually [69] and on a site-specific basis [70]. Initial research exploring experimental gingivitis in humans [71,72] indicated that the onset as well as severity of the gingivitis to the dental plaque accumulation varied noticeably amongst individuals. The discrepancies, however, were ascribed to quantitative plaque differences (differing plaque formation) or qualitative plaque differences (distinct microbial species in the plaque biofilm) [73,74]. Thus, the intensity of the periodontal inflammation may be a single trait [75] and predisposition to PD may also be influenced by host genetic factors [76,77,78].

There is no single host factor that has been recognized as the principal reason for vulnerability to PD. The fact that inflammatory mediators such as IL1, tumor necrosis factor, and prostaglandin E2 levels link with the periodontal destruction [79,80] and may exacerbate the inflammation [62] advocated that individuals forming high levels of these mediators will demonstrate greater detrimental loss of tissue. Reduced polymorphonuclear leukocyte numbers or activity can further hasten and worsen tissue destruction [81]. Many medications, including phenytoin (anti-epileptic), nifedipine (calcium channel blocker), and cyclosporine (immunosuppressant), can induce gingival hypergrowth and hence alter pre-existing periodontal infection [82]. Alterations in hormonal levels, such as estrogen, may increase gingival inflammation, but do not typically increase CP susceptibility [83]. The hormonal changes associated with menopause have been related to osteoporosis; however, the relationship between this condition or estrogen insufficiency and PD susceptibility is uncertain. Lastly, immunosuppression (both medication-induced and disease-induced) may increase the risk of periodontal tissue loss [84]. Indeed, a weakened immune system causes impaired host reactions to pathological infections, leading to more aggravated disease-induced injury and increased inflammatory response. Unlike the present-day knowledge on adaptive immunity, no immunoglobulins or lymphocytes have been clearly interconnected to a PD higher susceptibility [85]. 

### 2.4. Impact of Genetics and Epigenetics 

In a few studies, the genetical role in CP has been explored. A research on siblings who did not develop severe CP despite not having routine dental treatment revealed that hereditary factors may be at the root of the less severe types of PD [49,86]. Several genes are most likely implicated in CP, and CP genotypes may differ between persons and cultures. The polymorphism of genes implicated in cytokine production have received a lot of interest [87], although none of the single nucleotide polymorphisms have been observed repeatedly [53,88]. 

Although family studies can provide evidence for familial aggregation, they cannot differentiate between environmental and genetical effects since environmental variables can also influence gene expression. Epigenetic modifications influence gene expression patterns by methylation or acetylation of DNA bases or chromatin alterations impacting the readability of the genetic code [89], although the epigenetic processes involved in the regulation of inflammatory and anti-inflammatory genes remain unknown [90]. Epigenetics is a relatively new idea in CP research, and it has the potential to improve our understanding of the factors of susceptibility and population variance, as well as provide a connection between genetics, PD phenotypes, and the environment.

## 3. Regenerative Mechanisms of Periodontal Tissues

**Figure 3:**
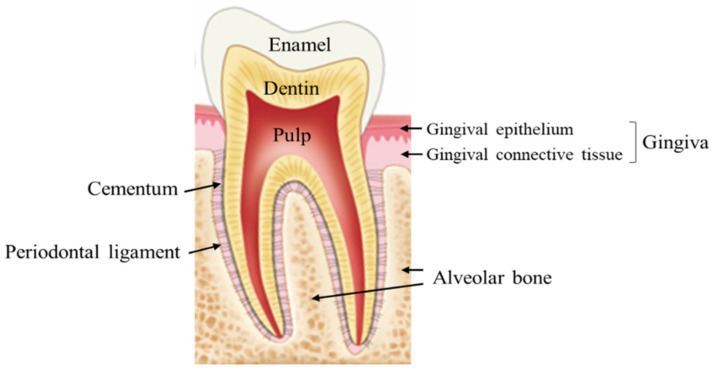
Diagrammatic demonstration of the periodontium encompassing the intact periodontal structures. The figure was adapted from Cho et al. (2021) [99].

Understanding the anatomy and physiogenesis of the periodontium provides a firm foundation for understanding the process of tissue deterioration and promoting the creation of more effective cell-based treatment modalities [91]. The periodontium, which supports and secures the teeth in their alveolar sockets, has a well-organized structure and may be separated into four major important components: gingiva, RC, PDL, and AB (Figure 3) [91,92]. The periodontal complex is the primary component of this apparatus and is hierarchically structured into the final three components (i.e., AB-PDL-RC) [93] and characterized by PDL fibers inserted into the RC and surrounding AB. Because of this multi-tissue complex’s interfacial linkage, the periodontium may support and maintain the teeth by dispersing and absorbing stresses, as well as act as a barrier to guard against various invading microbial pathogens [91,94,95,96]. Therefore, the periodontal complex is regarded as the foundation of the periodontal structure and performs critical functions in healing and sustaining proper tissue function. Because of the many tissue types and intricate structures involved, full regeneration of the periodontal complex is exceedingly difficult to achieve with local administration of stem cells alone [97,98]. Researchers postulate that the periodontal complex regeneration can obtain assistance from unique cell-material designs, drawing inspiration from the anatomical structure of the periodontium, and have applied the notion of tissue engineering to reconstruct the natural periodontal apparatus.

### 3.1. Cementum Regeneration

The RC formed as a thin acellular layer around the root neck, with a thicker cellular cementum covering the lower portion of the root up to the apex [100,101,102]. In the early cementogenesis, Hertwig’s epithelial root sheath (HERS) cells were thought to release acellular cementum. Later, the dental follicle-derived cementoblast formed cellular and reparative cementum. Several illnesses, including periodontitis, commonly damage the acellular cementum. However, in a clinical setting, the regularity and quality of RC regeneration appeared to be inadequate. As it contributed mostly to the attachment function, the regenerated RC should ideally look like the acellular extrinsic fiber cementum (AEFC). The characteristic of the attachment function was questioned in most periodontal regeneration investigations since the newly generated RC was cellular intrinsic fiber cementum (CIFC) rather than the expected AEFC. In CIFC, the numerical density of inserting fibers was small, and interfacial tissue bonding appeared insignificant [100,101,102].

Various cementum-specific proteins have been demonstrated to induce the cementogenesis and osteogenesis in injured periodontal tissues [103]. Cementum attachment protein (CAP), cementum-derived growth factor (CDGF), and cementum protein-1 were among the proteins studied (CEMP1). They may activate numerous mitogenesis-related signaling pathways, raise cytosolic Ca2+ concentrations, trigger the protein kinase C cascade, and stimulate progenitor cell motility and preferred adhesion. These activities may result in the development of cementoblasts and osteoblasts, as well as the creation of a mineralized extracellular matrix similar to RC [103]. Indeed, adding CEMP1 to 3D PDL cell cultures boosted alkaline phosphatase (ALP) specific activity by a factor of two and stimulated the cementogenic and osteogenic markers, resulting in the novel tissue formation resembling RC and AB [104].

Cementoblast progenitors were derived from stem cells in the gingival, PDL, and AB [105], which produced cementum-specific markers and cementum-like mineralized nodules in vitro [106]. Certainly, PDLSCs, DFSCs, and ADSCs were able to develop into cementoblasts and repair the periodontal tissues in vivo to construct RC-like tissue, PDL fibers, and periodontal blood vessels regeneration [107,108]. DFSCs were mixed with treated dentin matrix (TDM) and implanted into the dorsum of mice subcutaneously in another investigation. Histological analysis indicated a whirlpool-like orientation of the DFSCs in many layers that were positive for ALP, integrin 1, collagenase I (COLI), fibronectin, and ALP, indicating the production of a rich extracellular matrix. TDM has the potential to induce and promote DFSCs in the development of new RC-PDL complexes and dentin-pulp-like tissues, signifying effective RC regeneration [109]. As a result, utilizing these stem cells for periodontal abnormalities may be an efficient method for RC regeneration [110]. 

Furthermore, co-cultivating different cells may increase cementoblast development. The biological effects of developing apical tooth germ cell conditioned media (APTG-CM) on cementogenesis and PDLSCs differentiation were examined. The PDLSCs were grown with APTG-CM and displayed cementoblast lineage traits. Morphological alterations, increased ALP activity, increased proliferation and the expression of cementum-associated genes and mineral nodules were all observed. The induced PDLSCs displayed tissue-regenerative potential and formed new RC and PDL-like structures following transplantation in vivo in an immunocompromised mouse model. This structure was made from a layer of RC-like tissues with PDL-like collagen fibers linked to the novel RC. In contrast, the PDLSC transplant control produced solely connective tissues [111]. As a result, APTG-CM revealed the potential to provide a cementogenic milieu and promote PDLSC differentiation into the cementoblastic lineage; hence boosting periodontal tissue engineering. 

The “cell sheet” approach was established by rearing cells in high confluency until they formed a cell sheet and created significant cell–cell contacts [112]. The cells in the DPSCs sheets were able to survive for at least 4 days while maintaining their stemness and osteogenic differentiation capability. As a result, DPSCs sheets might be used as a natural three-dimensional structure for treating AB defects [113]. Likewise, human PDL cells were planted on temperature-reliant culture plates in a complete medium supplement for 72 h before being transferred to an osteo-inductive culture medium for another 48 h to achieve over-confluency. The cells sheets and PGA sheets were retrieved afterwards by detaching them off the plates [114]. This procedure was performed two times more, resulting in the PDL cell sheets. The exposed tooth root surfaces were covered with three-layered PDL cell sheets sustained by PGA sheets. The lesion was then packed with macroporous beta-tricalcium phosphate (ß-TCP) to form a three-walled intra-osseous lesion. Only the PGA sheets were used in the control group. A histometric study revealed full periodontal regeneration with well-aligned collagen fibers joining the novel RC to novel AB at 6 weeks. In addition, PDL-like structures grew surrounding the new RC and AB, which were later shown to be collagen fibers [115]. The autologous cells produced in vitro were successful for tissue regeneration and had previously been used in various clinical studies, including periodontal therapy [116].

The outcomes of micro-nano-hybrid surface HA bioceramics (mnHA) on the proliferation, attachment, osteogenic and cementogenic differentiation of PDLSC were examined to explain the capability of cementogenic differentiation of stem cells [117]. The mnHA bioceramics increased ALP activity, cell proliferation, and the osteogenic and cementogenic markers such as CAP and CEMP1, runt-related transcription factor 2 (Runx2), ALP, and osteocalcin. Furthermore, the canonical wingless-type MMTV integration site family (WNT) signaling inhibitor dickkopf1 may suppress the stimulatory impact on ALP activity as well as osteogenic and cementogenic gene expression (Dkk1). These results advocate that mnHA can be a promising biomaterial for RC and AB repair. Nonetheless, further animal as well and human research is required to assess and validate the effectiveness in periodontal regeneration. As a result, cementum-related proteins were able to regulate periodontal stability and regenerate RC structures. 

Contemporary research found that the following new measures encouraged RC repair as well as regeneration: (1) differentiation of cementoblast progenitors, (2) co-culture with cytokines released by other particular cells, (3) cell sheet use, and (4) materials with custom-made micro-nano-hybrid surfaces. These techniques provide novel and improved therapeutic approaches for managing PD and generating periodontal regeneration.

### 3.2. Periodontal Ligament

To reduce occlusal pressures, the PDL fibers join the RC to the AB and secure the tooth in the alveolus. PDL renewal is a critical prerequisite for periodontal regeneration. The optimum consequence would be the well-aligned regenerated collagen fibers to be securely connected to the regenerated RC and the novel AB [118]. PDs have the potential to alter the cell biology of the diseased periodontium. Once injured, the periodontal apparatus has a restricted potential for regeneration that is dependent on the MSCs availability. Several MSC types survive and are responsible for tissue homeostasis, as well as providing as a basis of renewable progenitor cells to make other cells needed during adulthood. Furthermore, research has revealed that periodontal stem cells may be implanted into periodontal abnormalities with no deleterious immunologic/inflammatory repercussions.

As a result, efficient recruitment of locally produced viable precursor cells to the lesion site for tissue stability and successive differentiation into PDL, RC, and AB cells is vital for periodontal regeneration [119]. Novel PDL-like tissues were effectively produced after stem cells were delivered to the defect locations [120,121], including PDLSCs [122,123], BMMSCs [29], ADSCs [124], and iPSCs [125]. PDLSCs were grown, osteogenically stimulated, and planted on a biphasic calcium phosphate scaffold (BCP) [61]. The scaffolds were then planted with PDLSCs and implanted. At 3 months, the results demonstrated excellent periodontal regeneration, comprising novel AB development and PDL with restructured and regenerated collage fibers incorporating into surrounding RC and AB at the appropriate position, as well as plentiful blood vessels. As a result, PDLSC-seeded scaffolds appeared to be a potential technique for periodontal regeneration. 

Several implantations of BRGs into periodontal defects, however, created a long junctional epithelium (LJE) but did not restore a true periodontal apparatus [126]. The creation of a LJE merely reduced PPD but had no effect on PDL fiber regeneration. In comparison, optimum periodontal tissue regeneration required well-arranged fibers that attached to surrounding novel RC and AB [127]. To increase the PDLSCs proliferation and the production of both PDL and AB, a barrier membrane was employed to preserve the distance between the defect and the tooth root [127]. Periodontal regeneration was shown histologically in animal investigations [126]. As a result, GTR might direct soft tissue regeneration while preventing down-growth into the bony lesions, supporting periodontal healing [128].

Non-resorbable membranes were more likely to be prone to the oral environment, raising the possibility of post-operative contamination [129]. A large number of patients were accessible for the study of vertical clinical attachment level (CAL-V) growth at 1 and 10 years following GTR treatment. At 1 and 10 years, the non-resorbable barrier group acquired 3.4 ± 1.0 and 1.5 ± 1.2 mm CAL-V, respectively. At 1 and 10 years, the bioabsorbable barrier group acquired 3.3 ± 1.6 and 3.5 ± 2.5 mm, respectively [130]. Another study found that after 9 months, the mean PPD decrease for bioabsorbable sites was 5.2 ± 3.9 and 5.5 ± 3.0 mm for the non-resorbable group. The CAL increase at 9 months was 5.9 ± 3.3 mm for the resorbable group and 5.5 ± 3.4 mm for the non-resorbable group [131]. As a result, there was an insignificant difference at 9 or 12 months; nevertheless, 10-year monitoring revealed that the absorbable group achieved higher CAL-V than the non-resorbable group (2.34-fold). Consequently, gingival mesenchymal stem cells (GMSCs) and PDLSCs were first encapsulated in a new arginine–glycine–aspartic acid tripeptide (RGD)-coupled alginate microencapsulation system to assess bone regeneration capability [132]. The production of osteogenic markers ALP, Runx2, and osteocalcin was increased in vitro by the microencapsulation scaffolds. In immunocompromised mice, critical-sized calvaria defects (5 mm) were produced, and the cell–RGD–alginate complex was implanted. At 2 months, new PDL fibers and AB were effectively produced [132,133]. Second, for periodontal tissue engineering applications, a combination of polylactic-co-glycolic acid (PLGA), chitosan, and silver nanoparticles was studied [134,135]. In vitro cell mineralization and osteogenic gene expression were achieved using this approach. Meanwhile, following planting into the lower jaw in vivo, the experimental group bone density was greater than the control group [136]. Next, a consequent collagen self-assembly approach was paired with mineral formation diffusion gradients to produce multiphasic collagen scaffolds with interconnectivity and macroporosity between the layers [137]. Mineralization occurred in the scaffold layers, with mineralized collagen fibrils including intrafibrillar and orientated minerals resembling bone. Furthermore, non-mineralized fibrils were placed into the mineralized layer to provide mechanical interlocking and consistency. As a result, these absorbable materials may be able to eliminate the necessity for a second operation as well as the hindered wound healing.

Moreover, a study examined the result of an electrospun nanofibrous membrane scaffold fabricated using the biomimetic fish collagen/bioactive glass/chitosan composite for periodontal regeneration [138] and reported an outstanding hydrophilicity and a moderately high tensile strength. The composite membrane outperformed the pure fish collagen membrane in terms of antibacterial activity against *Streptococcus mutans*. In vitro, the composite stimulated cell proliferation and osteogenic gene expression in PDLSCs. It also increased the expression of Runx2 and osteopontine. Animal experiments confirmed the capacity of this material to stimulate PDL and new AB development in class II (Glickman’s) furcation lesions. This new composite membrane possesses high macroporosity and surface area to facilitate cell to cell and cell to matrix interactions, as well as suitable antibacterial capabilities, tensile strength, and osteogenic qualities to promote periodontal regeneration.

### 3.3. Alveolar Bone

Periodontitis is characterized by AB loss. Periodontitis and AB loss can be caused by pathogenic biofilm microbes, hereditary causes, and environmental factors including cigarette smoking. The loss of supporting AB surrounding a tooth causes tooth mobility and displacement, ultimately leading to tooth loss [1]. Numerous approaches, such as BRGs [5,6,7,8,139], scaffolds [140], stem cells [141], and growth factors [9,10], have been developed to improve the osteogenesis process. BRGs are usually classified into four types [93,142]. To begin, autogenous bone transplants are often considered the “gold standard” in BRGs [143]. Clinical applications revealed the formation of new AB and periodontal connective tissue attachment [144]. Next, tissue banks provide a variety of allogeneic BRGs [145], such as demineralized freeze-dried bone allografts (DFDBAs) and freeze-dried bone allografts (FDBAs). AB fillings of 1.3–2.6 mm were achieved in clinical studies. DFDBAs produced significantly more vital bone (38.4%) than DFDBAs (24.6%) [146]. Third, xenografts, such as Bio-Oss [147], have been employed. One study looked at the results of using titanium mesh with Bio-Oss to augment localized alveolar ridges. A radiographic study revealed a 3.7 mm buccolabial ridge and 2.9 mm vertical augmentation, with histomorphometry revealing that 36–37% of the treated area contained novel AB [148]. Finally, synthetic alloplastic biomaterials such as hydroxyapatite (HA) [149,150,151], TCP [152], a calcium-layered-polymer of polymethyl methacrylate and hydroxyethyl methacrylate [153], and bioactive glass [154] have been produced. For bone regeneration applications, injectable and absorbable scaffolds have been produced [155,156]. Calcium phosphate cements (CPCs) were made from powders combined with a liquid to make a paste [157,158]. Through a dissolution–precipitation reaction [159], the paste can be inoculated into the bony lesion and hardened in situ to create a scaffold. Cell implanting onto the porous CPC scaffold resulted in a low seeding effectiveness and average cell penetration [160]. Because the mixing pressures, ionic exchanges, and pH fluctuations during the CPC paste setting were damaging to the cells, it was not possible to directly add the cells to the CPC paste. As a result, a resorbable and injectable alginate-microfibers/microbeads (Alg-MF/MB) stem cell delivery system was designed to preserve the encapsulated stem cells throughout CPC paste mixing and injection [161]. Microbeads degraded after 72–96 h, releasing the encapsulated cells [162,163]. In a recent investigation [164], six different stem cells were encapsulated in hydrogel MF/MB inside an injectable CPC: (1) human bone MSCs, (2) DPSCs [165,166], (3) hUCSCs, (4) MSCs-ESCs, and iPSCs-MSCs derived from (5) bone marrow and (6) foreskin at 1 week; all of the cells above proliferated and osteodifferentiated well, with strong expressions of osteogenic genes. Cell-synthesized bone matrix minerals increased with culture period, demonstrating outstanding bone regeneration potential comparable to gold-standard human bone MSCs [167]. Following that, when the human bone MSC-encapsulated Alg-MB-CPC paste was implanted into a bony lesion for regeneration in animals, the construct demonstrated a powerful potential for novel AB production. At 3 months, an osseous bridge was established in the AB defect, which was three times more than the control group [163]. As a result, the absorbable, injectable, load-bearing stem cell-MF/MB-CPC construct appeared favorable for cell delivery to significantly improve AB regeneration in periodontal repairs [159]. Other hybrid polyethylene glycol-co-peptide hydrogels might be tuned to in situ gelation needs, providing a possibility for injectable usage. This new hydrogel has intriguing uses in endogenous regeneration, indicating an additional modern treatment method [168].

It was also critical to develop vascularization in periodontal regeneration [16,169,170]. To offer pre-vascularization to CPC scaffold, a tri-culture system containing human umbilical vein endothelial cells, iPSCs-MSCs, and pericytes was recently developed [169]. In vitro, vessel-like structures were developed in the co-cultured and tri-cultured groups. Furthermore, osteogenic and angiogenic marker expression, and bone matrix mineralization, were significantly increased. A calvarial defect model in animals was employed, and following 3 months, the tri-culture group developed more novel bone (45%, 4.5-fold) and vessel density (2.5-fold, 50%) than the control. The site proportion of newly created bone as well as blood vessel density in the tri-culture constructions were about 1.2 and 1.7 times higher than in the co-culture group, respectively [163]. Furthermore, innovative nanomaterials have been produced in collaboration with various scaffold materials and biologics to improve periodontal regeneration [121,170,171,172]. Bone MSCs were combined with bone morphogenetic protein (BMP)-7, implanted on nHA/PA porous scaffolds, and subsequently seeded in a lower jaw deficiency model in vivo [173,174]. BMP-7-transfected MSC scaffolds revealed a quicker reaction as compared to the MSCs and pure nHA/PA scaffolds. As a result, this investigation demonstrated the significance of the factors and cells in aiding bone repair. Gold nanoparticles (GNPs) were added into CPC in different research [175]. On CPC, this enhanced cell adhesion, proliferation, and osteogenic induction. Furthermore, the released GNPs were internalized by DPSCs, resulting in increased expression of ALP at 1- week (3-fold), osteogenic gene at 2 weeks (2–3-fold), and cell mineral at 2 weeks (5-fold). As a result, nanoparticles had potential for modifying scaffolds and collaborating with different bioactive materials to improve the bone regeneration in periodontium.

## 4. Dental and Non-Dental Stem Cells for Regenerating Periodontal Apparatus

### 4.1. Stem Cells from Dental Tissues

**Figure 4:**
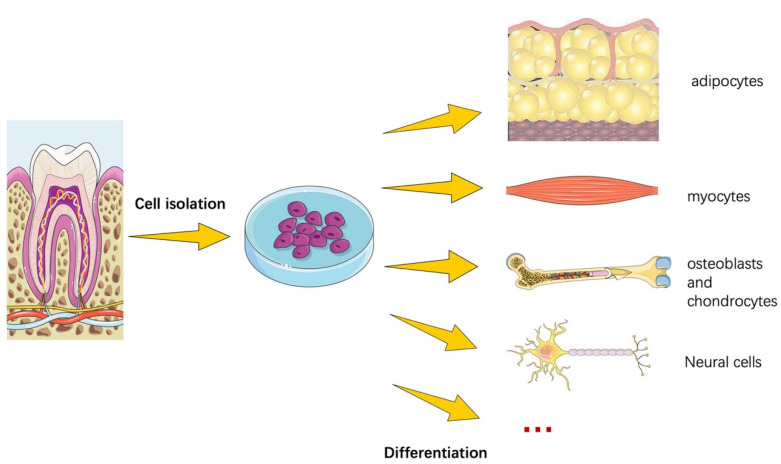
DSCs, similar to members of the MSCs family, have the capacity to differentiate into several lineages. Experiments have shown that given the right conditions, DSCs may develop into bony tissues such as osteoblasts, adipose tissues such as adipocytes and chondrocytes, and nerve and neuronal tissues. The figure was adapted from Wang et al. (2019) [181].

**Figure 5:**
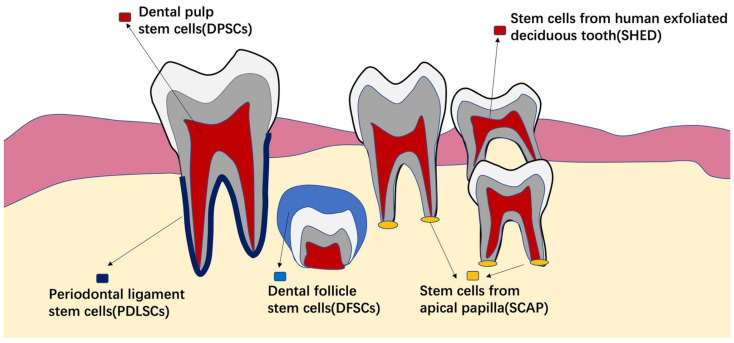
DSCs, which include DPSCs, PDLSCs, DFSCs, SHEDs, and SCAPs, are classified into numerous groups depending on their origin. The dental pulp is used to isolate DPSCs. PDLSCs are a type of cell found in the PDL. SHEDs are formed when deciduous teeth are exfoliated. DFSCs are obtained from the dental follicle of a tooth that has not yet erupted. The apical papilla is used to isolate SCAPs. The figure was adapted from Wang et al. (2019) [181].

Dental stem cells (DSCs) are multipotent, self-renewable MSCs [176]. DSCs can be odontogenic, dentinogenic, cementogenic, osteogenic, chondrogenic, adipogenic, myogenic, or neurogenic [177] (Figure 4). DSCs (Figure 5) are easy to find as they can be discovered in the human body at any age. Additionally, cryopreservation has little effect on their characteristics [178]. These traits make them approachable and simple to use [179]. Moreover, stem cells that can be isolated from various oral tissues have emerged as attractive sources for bone as well as dental regeneration, primarily due to their ease of accessibility [180]. 

DPSCs were among the first DSCs to be discovered. They are widely available since they may be produced from human dental pulp. DPSCs are particularly appealing for a variety of reasons. For starters, DPSCs are located in dental pulp and may be harvested using simply one removed tooth, especially wisdom teeth and periodontally damaged teeth. It has also been proposed that they may be derived from inflamed tooth pulp [182] and yet exhibit the ability to develop into osteoblast-like cells. Second, DPSCs are related to periodontal stem cells in terms of origin, antigenic pattern, and differentiation lineages [33,180,183]. Furthermore, they can develop into cardiomyocytes, neuron cells, adipocytes, melanoma cells, corneal epithelial cells, and insulin secreting Beta cells [184]. Additionally, they interact readily with other bioactive materials [183,185].

PDLSCs have been regarded as an attractive treatment approach for periodontal regeneration as they may be quickly retrieved by non-invasive techniques following simple tooth extraction. They may also be cultivated, and have osteogenic, chondrogenic, and adipogenic capabilities [186], as well as immunosuppressive properties comparable to BMMSCs and DPSCs. They are capable of forming a RC/PDL complex-like structure. 

DFSCs may function as PDLSCs precursor cells. PDLSC proliferation, osteogenic and adipogenic differentiation are all enhanced to varying degrees by DFSCs. In vitro co-culture with DFSCs enhances the cell layers and extracellular matrix of PDLSCs sheets [187]. However, there is currently a scarcity of scientific data regarding this cell type.

Because SHEDs are extracted from deciduous exfoliated teeth, they are easier to access utilizing non-invasive techniques. SHEDs have a greater proliferation rate and immunomodulatory characteristics, comparable to BMMSCs, that are more challenging to achieve. They can develop into osteoblasts [188] and have the ability to regulate T cells, macrophages, and dendritic cells [189]. When a study related the “stemness” of SHEDs to that of DPSCs and BMMSCs, they discovered that SHEDs had a greater proliferation rate and greater expression of cell proliferation and extracellular matrix genes than DPSCs and BMMSCs. As a result, SHEDs is a promising option for periodontal regeneration [190]. 

SCAPs are associated with root development. Their existence in the apical papilla of developing roots has been proposed as a probable justification for how juvenile teeth with necrotic pulps may generate roots. SCAPs are also infection-resistant [191], which may explain why apexogenesis has been found even in the presence of apical periodontitis. Despite their difficulty in terms of collection, they are a promising tool for regenerative operations since they have the potential for multi-lineage differentiation [192].

Dental stem cells have excellent potential for osteogenesis and may be successfully used to replace usually used BMMSCs. Some sources such as DFSCs, SHED, SCAP, and others need evaluation beyond animal studies and necessitates human studies for clinical applications. More randomized clinical trials are advisable to standardize the clinical procedure and strengthen the claim of dental stem cells as potential replacement of other non-dental-tissue-based stem cells [177].

### 4.2. Stem Cells from Non-Dental Tissues

BMMSCs were the first MSCs found and have been extensively studied in animal models. They have differentiated into osteogenic, adipogenic, chondrogenic, and myogenic cells. The main limitation of BMMSCs is the discomfort associated with bone marrow harvesting, as well as the restricted quantity that can be harvested. BMMSCs have the ability to develop into ameloblast-like cells [193,194] and periodontal tissue cells, as well as improve periodontal regeneration [195,196]. Interestingly, BMMSCs may be employed for tooth regeneration in addition to periodontal regeneration since they may upregulate the expression of odontogenic genes and contribute to new tooth development following recombination with embryonic oral epithelium [197]. ADSCs are numerous stem cells produced from adipose tissues. ADSCs may be expanded in vitro and have demonstrated osteogenic, chondrogenic, adipogenic, and neurogenic differentiation in a variety of experimental situations. Because ADSCs may be extracted in large numbers from either liposuction or subcutaneous adipose tissue pieces, the harvesting approach is less invasive than that employed for BMMSCs. As a result, they have been widely employed in regenerative medicine.

ESCs are discovered in human blastocysts. They have a remarkable ability to differentiate, since they can grow into practically all cell lineages [198]. It has been demonstrated in the context of periodontal research that ESCs may develop into odontogenic and periodontal cell lineages, particularly when co-cultured with PDLSCs or embryonic oral epithelial cells [199,200]. Because extracting such cells may result in the killing of human blastocysts, ethical issues have limited their use for periodontal regeneration. Furthermore, in addition to their limitless potential, they have exhibited significant negative consequences such as tumors and undesired immunological reactions. Since their discovery in 2006, iPSCs have sparked significant interest in regenerative medicine [201]. They are a form of pluripotent stem cell which may be created from a somatic cell directly. They have the capacity to reproduce forever and develop into every other cell type in the body. Dental cells, such as DPSCs, PDLSCs, SHEDs, and SCAPs, have recently been effectively converted into iPSCs [202,203], and iPSCs have been studied for periodontal regeneration.

MSCs are thought to be an appealing tool for tissue regeneration because they have a significant immunomodulatory potential. Numerous in vitro studies suggest that the influence of dental MSCs on immune cells may be affected by a variety of parameters, including the experimental context, MSC tissue source, and type of immune cell preparation. Most studies have shown that the immunomodulatory activity of dental MSCs is strongly upregulated by activated immune cells. MSCs exert mostly immunosuppressive effects, leading to the dampening of immune cell activation. Thus, the reciprocal interaction between dental MSCs and immune cells provides an elegant mechanism that may contribute to tissue homeostasis and the evolution of inflammatory diseases. Although dental MSCs’ immunomodulatory capacity has been widely studied in vitro, their effect in vivo remains unknown. Several investigations have found that MSCs derived from inflammatory dental tissues have a weakened immunomodulatory potential. Furthermore, the expression of several immunomodulatory proteins is increased in periodontal disease and has been linked to disease severity. Immunomodulation based on MSCs may be important in the regeneration of various dental tissues. As a result, immunomodulation-based techniques have the potential to be a very promising tool in regenerative dentistry [204].

## 5. Umbilical Cord Stem Cells

**Figure 6:**
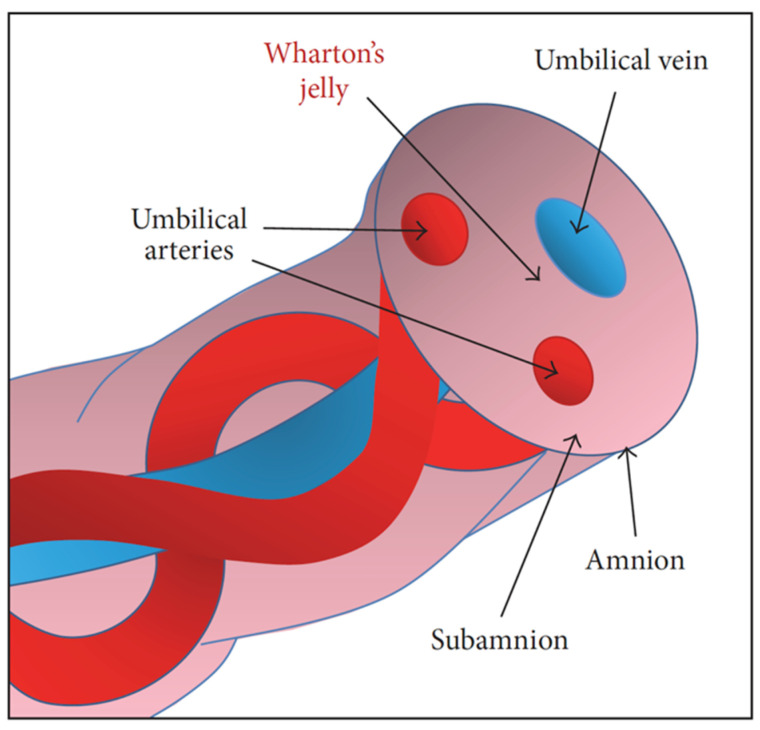
Diagrammatic representation of human umbilical cord. The figure was adapted from Szepesi et al. (2016) [206].

The umbilical cord is made up of a vein and two arteries that are encircled by Wharton’s jelly and coated by a basic epithelial membrane (Figure 6). Wharton’s jelly shields the blood vessels, keeps them from clogging, and keeps the cord flexible [205].

Human umbilical cord stem cells (hUCSCs) were employed in clinical practice for the first time in 1988, when Eliane Gluckman effectively transplanted cord blood cells into a 6-year-old child with Fanconi anaemia, utilizing hematopoietic stem cells (HSCs) [207]. The world’s first umbilical cord blood (UCB) bank was created in 1992. It is vital to note that hUCSC grafts are of higher quality in comparison to bone marrow grafts. Furthermore, the number of hUCSCs essential for an efficacious transplant is 10 times less than the bone marrow cells and peripheral blood cells. Indeed, the HSCs concentration of 80–120 mL of blood from a single umbilical cord is similar to 1200 mL of bone marrow [208,209]. Interestingly, evidence suggests that umbilical cord contains not only hematopoietic progenitors, but also a variety of other stem/progenitor cells such as MSCs, very small embryonic-like stem cells (VSELs), endothelial progenitors, unrestricted somatic stem cells (USSCs), and epithelial stem cells [210].

### 5.1. Differentiation, Proliferation, Pluripotency, and Senescence Characteristics of Human UC-MSCs Compared to Other Stem Cells

To maximize the recovery of all stem cell types, it is desirable to retain both UCB and cord tissue. Umbilical cord can be used to separate hematopoietic, epithelial, and mesenchymal cells. The UCB arteries, intra- and peri-vascular zones, subamniotic zone, and amniotic epithelium are all important stem cells sources. Indeed, HSCs are present in UCB and may be used to generate a variety of blood cells. Furthermore, MSCs were recovered effectively from Wharton’s jelly, amniotic fluid, and membrane and cord lining [211], whereas epithelial cells were identified from the interior and exterior umbilical cord layers [212]. Table 1 illustrates the many cell types extracted from hUCSCs.

An in vitro study by Kim et al. (2017) described and compared MSCs from the PDL, umbilical cord, and adipose tissue, which were reasonably straightforward to acquire with few ethical issues regarding their procurement. UC-MSCs grew the fastest among MSCs extracted from the three distinct tissues. The indoleamine 2,3-dioxygenase and cyclooxygenase-2 pathways were revealed to suppress the proliferation of activated peripheral blood mononuclear cells (PBMCs) to a comparable extent in all three types of MSCs. They were also found to limit PBMC proliferation utilizing HLA-G, which was most prevalent in UC-MSCs. UC-MSCs, unlike the other two kinds of MSCs, displayed limited HLA-DR expression after activation, suggesting that they represent little danger of starting an allogeneic immune reaction when delivered in vivo. These qualities, as well as the simplicity of collecting and the lack of ethical issues about their usage, imply that UC-MSCs are potential MSC therapeutic candidates [213].

However, when GMSCs were compared to UC-MSCs, MSCs derived from gingiva have a higher proliferation rate and population doubling time than UC-MSCs. In contrast to UC-MSCs, immunofluorescence studies revealed the presence of pluripotency markers OCT-4 and NANOG in the cytoplasm of GMSCs, which was confirmed by Western blot. The mechanical properties of GMSCs, such as modulus of elasticity, are comparable to those of UC-MSCs, but surface roughness was found to be lower in GMSCs, suggesting that GMSCs have a greater adhesive property to the extracellular matrix. The neuronal differentiation rate of GMSCs and UC-MSCs differs only marginally; both cells expressed positivity for several neuronal lineage markers. Immunofluorescent results suggesting Tuj1 and neurofilament (NF) showed significantly higher expression in GMSCs than UC-MSCs statistically. However, Map-2, TAU, and GFAP (neural lineage) of UC-MSCs showed significantly higher expression compared to GMSCs. Hence, GMSCs constitute an autogenous source of MSCs, which are simple to acquire with the least morbidity, multipotent in nature with acceptable biological and mechanical qualities, presumably a perfect choice for clinical applications [214].

Another laboratory-based study conducted by Wang et al. (2018) assessed and compared the regenerative ability of UC-MSCs and BMMSCs to see if UC-MSCs could be exploited as a new cell type for bone regeneration. The proliferation and osteogenic potential of BMMSCs and UC-MSCs were examined in vitro. The bone regeneration potentials of BMMSCs and UC-MSCs were assessed by studying their ability for ectopic bone formation in a mouse model as well as their efficiency in a rat model of tibia bone deficiency. Radiological, histological, and immunohistochemical investigations were used to determine the amount of bone regeneration. The findings demonstrated that UC-MSCs have a strong osteogenic differentiation capability, similar to BMMSCs, and that UC-MSCs can contribute to bone and blood vessel regeneration. Furthermore, no significant variations in the bone regeneration effect were found between BMMSCs and UC-MSCs [215].

A different study, on the other hand, evaluated the baseline osteogenic potential of UC-MSCs and BMMSCs, and revealed opposite results. Different TGF-1 and BMP-2 signaling pathway inhibitors/activators, as well as the secretome of differentiating BMMSCs, were investigated. Cytochemical staining, as well as gene expression and proteomic analyses, demonstrated that UC-MSC had a low level of osteogenic commitment. However, stimulating the BMP-2 pathway with BMP-2 plus tanshinone IIA, as well as adding extracellular vesicles or protein-enriched preparations from developing BMMSCs, increased UC-MSC osteogenesis [216].

Furthermore, a study by Das et al. (2021) compared the senescence and proliferative characteristics of MSCs derived from dental pulp and umbilical cord. Their proliferation capability and replicative senescence at different passage numbers were examined in this work (i.e., P2 and P6). At P6, intracellular reactive oxygen species generation in DPSCs was considerably lower than in UC-MSCs (*p* < 0.001). ß-gal expression was found considerably lower in DPSCs culture than in UC-MSCs culture at P6 (*p* < 0.001). According to the findings, the source of stem cells determines both MSCs proliferative capacity and replicative senescence. As a result, this work will pave the way for future investigations into choosing acceptable stem cell sources for regenerative therapeutic applications [217].

### 5.2. Umbilical Cord Stem Cell Applications

hUCSCs treatment has enormous promise for curing a variety of human ailments. Table 2 below depicts the many clinical uses of umbilical cord stem cells [205].

## 6. Role in Periodontal Regeneration and Potential Clinical Applications

A laboratory-based study by Yu et al. (2013) compared the features of MSCs from the Wharton’s jelly part of the umbilical cord and PDLSCs for periodontal regeneration. PDLSCs were discovered to have more osteo-/dentinogenic, adipogenic, and chondrogenic differentiation capacity than Wharton’s jelly MSCs. A microarray study revealed that when Wharton’s jelly MSCs were compared to PDLSCs, 903 genes were significantly downregulated and 726 genes were significantly upregulated. They discovered that various genes may be related to MSCs properties based on microarray data. Further bioinformatic research revealed that transforming growth factor-ß (TGF-ß) and wingless-type MMTV integration site family (WNT) signaling pathways, as well as many genes such as signal transducer and activator of transcription 5B (STAT5B) and integrin ***α***4 (ITGA4), may play important roles in MSCs. These findings suggest that Wharton’s jelly MSCs have much lower differentiation capacity than PDLSCs, and therefore unmodified Wharton’s jelly MSCs may not be effective seeding cells for periodontal regeneration. This also aids in the understanding of MSC differentiation mechanisms and the identification of essential variables that promote Wharton’s jelly MSC-mediated periodontal regeneration [238].

Another in vitro study examined the proliferation, development, and adhesion of cultured UC-MSCs alone or in combination with basic fibroblast growth factor (FGF) on healthy and periodontally compromised tooth surfaces. A total of four groups were made: Group 1: healthy; Group 2: periodontally compromised; Group 3: healthy with FGF; and Group 4: periodontally compromised with FGF. FGF was added to the surface at a concentration of 8 ng/mL, followed by the incubation of cultured UC-MSCs on the scaffolds. On the 2nd and 3rd weeks, scanning electron microscopy examinations were undertaken to analyze the morphology and proliferation of cells adhered to the tooth surface. Cultured UC-MSCs adhered to the tooth root scaffold. From the 2nd to the 3rd week, all groups displayed a considerable increase in cell attachments. In comparison to the groups that did not have FGF, the groups that did had a considerable increase in cell attachment. The cells in all groups exhibited an increase in flat cells from the 2nd to the 3rd week, suggesting increasing cell maturity. Cell maturity was lower in periodontally infected groups than in healthy groups. The FGF-added groups had more mature cells as compared to the non-FGF-added groups. Human UC-MSCs have the ability to develop into cells that can bind to tooth root surfaces. Incubating UC-MSCs with FGF improved their proliferation and adhesion to root surfaces. These findings by George et al. (2015) suggest that involvement of UC-MSCs in periodontal regeneration can be investigated further [239].

Furthermore, a study explored the pluripotent and proliferative capacity of osteogenic differentiation of human UC-MSCs in improving periodontal repair. To imitate periodontal tissue recovery in vivo, dentine and pre-differentiated or undifferentiated cells were implanted subcutaneously into immunodeficient animals. The findings revealed that human UC-MSCs were easily obtained and expressed a variety of MSC markers. During osteogenic differentiation, the expression of stemness markers reduced significantly. Li et al. (2014) discovered that the osteogenic process could be initiated and identified at 1 week after investigating several time periods. In vivo, pre-differentiated UC-MSCs demonstrated an improved capacity to generate RC-like deposits surrounded by fibroblast-like tissue on the dentine surface. Finally, the capacity to generate RC-like tissue and the capability for proliferation and differentiation imply that UC-MSCs are interesting candidates as a source of MSCs for periodontal repair following auto-transplantation of teeth [240].

A patient with multiple gingival recession (Miller’s Class II) was chosen for therapy utilizing human UC-MSCs in conjunction with bone regeneration polycaprolactone (PCL) scaffold in a clinical study by Kadam et al. (2019). Clinical indicators such as gingival recession, PPD, CAL, and keratinized gingiva width were measured at baseline and 6-months after surgery. Six months after surgery, there was a considerable decrease in gingival recession, indicating more than 80% root coverage; thus, demonstrating the efficacy of bone regenerating PCL scaffold in conjunction with MSCs from the Wharton’s jelly part of the human umbilical cord [241].

Shang et al. (2017) compared the regenerative ability of PDLSCs to that of human UC-MSCs for periodontal regeneration. Comparing UC-MSCs to PDLSCs revealed traits such as multi-differentiation capacity and anti-inflammatory potential. Cell aggregates (CA) were created utilizing UC-MSCs and PDLSCs, respectively. The regeneration potentials of PDLSCs-CA and UC-MSCs-CA were then tested in an animal inflammatory periodontal defect model using ß-tricalcium phosphate bioceramic (ß-TCP). PDLSCs were shown to have more osteogenic differentiation capability than human UC-MSCs. Meanwhile, UC-MSCs secreted more extracellular matrix-related genes, including fibronectin, integrin β, and collagen type I, and had better anti-inflammatory properties, i.e., higher expression of TGF-β than PDLSCs. Both groups demonstrated soft and hard periodontal tissue regeneration in the presence of inflammatory periodontitis in rats. Additionally, both groups demonstrated more newly produced AB and PDL in comparison to the non-cell treated group. Furthermore, insignificant variations in regeneration boosting effects were detected between PDLSCs and UC-MSCs, suggesting that UC-MSCs had similar stimulating effects on periodontal regeneration and may be applied as novel cell sources for periodontal regeneration [242].

An animal study by Sun et al. (2020) investigated the effect of UC-MSCs mixed with bone collagen particles on the healing of AB cleft defects in rabbits. By removing the anterior teeth of the maxillary jaw, bone collagen particles mixed with UC-MSCs were placed in the defect site to create an animal model of AB clefts. Following 3 months post-surgery, blood biochemical analysis was completed. Gross examination and micro-focus computerized tomography studies of skull tissues were performed. Histological as well as immunohistochemical staining of tissues was performed. Six months following surgery, the experiments were repeated. Biocompatibility was observed between bone collagen particles and UC-MSCs. Bone collagen particles coupled with UC-MSCs were much more effective than bone collagen particles only in promoting bone repair and regeneration; thereby providing a simple, quick, and effective strategy for filling a bone defect area and treating AB alveolar cleft lesions [243].

Moreover, the role of UC-MSCs in regenerating teeth has been investigated in an animal study by Chen et al. (2015). The study revealed that UC-MSCs may be stimulated into odontoblast-like cells. Induced UC-MSCs produced dentin-associated proteins such as dentin sialoprotein (DSP) and dentin matrix protein-1 (DMP-1) at levels comparable to native pulp tissue cells. Furthermore, DSP- and DMP-1-positive calcifications were found following UC-MSCs grafting in vivo. These results revealed that UC-MSCs have the ability to develop into odontoblast-like cells with typical dentin-like matrix deposition in vivo, indicating the use of UC-MSCs as a therapeutic source of cells for tooth regeneration [244].

## 7. Challenges and Issues Pertaining to Human UC-MSCs

The use of human UC-MSCs in regenerative clinical treatment holds much merit. However, it is not without challenges and concerns, especially as UC-MSCs transplantation is yet to be approved by the US Food and Drug Administration (FDA). It is still not fully understood whether there is a need for cross-matching where MSCs transplantation is concerned as trials have reached different conclusions [245,246]. Moreover, the ethical concern is raised whereby although the umbilical cord is an unemployed organ, both the donor and the recipient have the right of informed consent. There is little known regarding MSCs therapy. There is no system in place yet to detect the potential risk in donors. Additionally, potential long-term associated risks of MSC transplantation have not been explored fully [247]. Whether the UC-MSCs should be differentiated in vitro into the desired tissue of choice prior to the transplantation or whether they can be transplanted into the recipient directly and be allowed to differentiate and graft in vivo remains unanswered. Whether improvements in functional outcome will be mediated through engraftment of differentiated tissue or through paracrine effects (as seen in autologous BMMSCs transplantation) is also unknown. Furthermore, in clinical trials, evaluating whether allogeneic UC-MSCs will engraft successfully in humans as they have hypoimmunogenic properties is a significant challenge [248]. 

## 8. Conclusion and Future Directions

From the above discussion, it can by summarized that human UC-MSCs possess a huge potential in therapeutic as well as regenerative applications, as evident by the literature presenting successful results. Nevertheless, the research on the applications of UC-MSCs on periodontal regeneration is very scant and still needs more laboratory-based and clinical studies for further validation. In terms of future perspectives, the application of human UC-MSCs for periodontal regeneration is very positive as a few studies discussed above showed beneficial results. Keeping in mind the advantages of human UC-MSCs, in the next era, they can be successfully used for periodontal regeneration in different hard tissue (intraosseous and furcation) and soft tissue (gingival recession) defects as used in other medical-based regenerative applications.

## Figures and Tables

**Table 1:** Various cells extracted from hUCSCs.

Hematopoietic stem cells
Epithelial stem cells
Mesenchymal stem cells
Endothelial progenitors
Induced pluripotent stem cells

**Table 2:** Clinical applications of hUCSCs.

Clinical Application	Main Diseases	Type of hUCSCs Applied
Treatment of hematological diseases	Sickle cell anemia [218]	HSCs
	Aplastic anemia [219]	HSCs
	Thalassemia [220]	HSCs
	Leukemia [221]	HSCs
Treatment of cardiovascular diseases	Buerger’s disease [222]	MSCs
	Dilated cardiomyopathy [223]	MSCs
	Stroke [224]	MSCs
Bone regeneration	Osteoporosis [225]	MSCs
	Congenital abnormalities, trauma, tumor resections, fractures as well as disorders such as arthritis	MSCs
Treatment of eyesight diseases	Diabetic retinopathy-associated neurodegeneration [226]	MSCs
	Traumatic optic neuropathy [227]	MSCs
Treatment of metabolic disorders	Type 1 diabetes [228,229]	MSCs
	Type 2 diabetes [230]	MSCs
Treatment of neurodegenerative and neurodevelopmental disorders	Parkinson’s disease [231]	MSCs
	Huntington’s disease [232]	MSCs
	Alzheimer’s disease [233]	MSCs
	Amyotrophic lateral sclerosis [234]	MSCs
	Autism [235]	MSCs
Wound healing	Burn injuries [236]	MSCs
	Chronic ulcers in diabetes [237]	MSCs
HSCs (hematopoietic stem cells); MSCs (mesenchymal stem cells)		

## Data Availability

Not applicable.

## References

[1] Pihlstrom B.L., Michalowicz B.S., Johnson N.W. (2005). Periodontal diseases. Lancet..

[2] Villar C.C., Cochran D.L. (2010). Regeneration of periodontal tissues: Guided tissue regeneration. Dent. Clin. N. Am..

[3] Nyman S., Gottlow J., Karring T., Lindhe J. (1982). The regenerative potential of the periodontal ligament: An experimental study in the monkey. J. Clin. Periodontol..

[4] Chen F.-M., Sun H.-H., Lu H., Yu Q. (2012). Stem cell-delivery therapeutics for periodontal tissue regeneration. Biomaterials.

[5] Shaikh M.S., Husain S., Lone M.A., Lone M.A., Akhlaq H., Zafar M.S. (2020). Clinical effectiveness of anorganic bovine-derived hydroxyapatite matrix/cell-binding peptide grafts for regeneration of periodontal defects: A systematic review and meta-analysis. Regen. Med..

[6] Shaikh M.S., Zafar M.S., Alnazzawi A. (2021). Comparing Nanohydroxyapatite Graft and Other Bone Grafts in the Repair of Periodontal Infrabony Lesions: A Systematic Review and Meta-Analysis. Int. J. Mol. Sci..

[7] Reynolds M.A., Aichelmann-Reidy M.E., Branch-Mays G.L., Gunsolley J.C. (2003). The efficacy of bone replacement grafts in the treatment of periodontal osseous defects. A systematic review. Ann. Periodontol..

[8] Shaikh M.S., Zafar M.S., Alnazzawi A., Javed F. (2022). Nanocrystalline hydroxyapatite in regeneration of periodontal intrabony defects: A systematic review and meta-analysis. Ann. Anat..

[9] Takeda K., Shiba H., Mizuno N., Hasegawa N., Mouri Y., Hirachi A., Yoshino H., Kawaguchi H., Kurihara H. (2005). Brain-derived neurotrophic factor enhances periodontal tissue regeneration. Tissue Eng..

[10] Zhou S., Sun C., Huang S., Wu X., Zhao Y., Pan C., Wang H., Liu J., Li Q., Kou Y. (2018). Efficacy of adjunctive bioactive materials in the treatment of periodontal intrabony defects: A systematic review and meta-analysis. BioMed Res. Int..

[11] Koop R., Merheb J., Quirynen M. (2012). Periodontal regeneration with enamel matrix derivative in reconstructive periodontal therapy: A systematic review. J. Periodontol..

[12] Annunziata M., Piccirillo A., Perillo F., Cecoro G., Nastri L., Guida L. (2019). Enamel matrix derivative and autogenous bone graft for periodontal regeneration of intrabony defects in humans: A systematic review and meta-analysis. Materials.

[13] Shaikh M.S., Pisani F., De Vito D., Lone M.A., Almasri M. (2021). Long-term Clinical Performance of Regeneration versus Conservative Surgery in the Treatment of Infra-bony Defects: A Systematic Review. J. Int. Acad. Periodontol..

[14] Kao R.T., Nares S., Reynolds M.A. (2015). Periodontal regeneration–intrabony defects: A systematic review from the AAP regeneration workshop. J. Periodontol..

[15] Avila-Ortiz G., De Buitrago J.G., Reddy M.S. (2015). Periodontal regeneration–furcation defects: A systematic review from the AAP Regeneration Workshop. J. Periodontol..

[16] Shaikh M.S., Zafar M.S., Pisani F., Lone M.A., Malik Y.R. (2021). Critical features of periodontal flaps with regard to blood clot stability: A review. J. Oral Biosci..

[17] Cortellini P., Tonetti M.S. (2015). Clinical concepts for regenerative therapy in intrabony defects. Periodontol. 2000.

[18] Gottlow J., Nyman S., Karring T., Lindhe J. (1984). New attachment formation as the result of controlled tissue regeneration. J. Clin. Periodontol..

[19] Cortellini P., Tonetti M.S. (2007). A minimally invasive surgical technique with an enamel matrix derivative in the regenerative treatment of intra-bony defects: A novel approach to limit morbidity. J. Clin. Periodontol..

[20] Cortellini P., Tonetti M.S. (2009). Improved wound stability with a modified minimally invasive surgical technique in the regenerative treatment of isolated interdental intrabony defects. J. Clin. Periodontol..

[21] Harrel S.K. (1999). A minimally invasive surgical approach for periodontal regeneration: Surgical technique and observations. J. Periodontol..

[22] Trombelli L., Farina R., Franceschetti G., Calura G. (2009). Single-flap approach with buccal access in periodontal reconstructive procedures. J. Periodontol..

[23] Aslan S., Buduneli N., Cortellini P. (2017). Entire papilla preservation technique in the regenerative treatment of deep intrabony defects: 1-Year results. J. Clin. Periodontol..

[24] Aslan S., Buduneli N., Cortellini P. (2020). Clinical outcomes of the entire papilla preservation technique with and without biomaterials in the treatment of isolated intrabony defects: A randomized controlled clinical trial. J. Clin. Periodontol..

[25] Hammarström L. (1997). The role of enamel matrix proteins in the development of cementum and periodontal tissues. Ciba Found. Symp..

[26] Trombelli L., Farina R. (2008). Clinical outcomes with bioactive agents alone or in combination with grafting or guided tissue regeneration. J. Clin. Periodontol..

[27] Kolios G., Moodley Y. (2013). Introduction to stem cells and regenerative medicine. Respiration.

[28] Yan X.-Z., Van Den Beucken J.J., Both S.K., Yang P.-S., Jansen J.A., Yang F. (2014). Biomaterial strategies for stem cell maintenance during in vitro expansion. Tissue Eng. Part B Rev..

[29] Du J., Shan Z., Ma P., Wang S., Fan Z. (2014). Allogeneic bone marrow mesenchymal stem cell transplantation for periodontal regeneration. J. Dent. Res..

[30] Yu N., Oortgiesen D.A., Bronckers A.L., Yang F., Walboomers X.F., Jansen J.A. (2013). Enhanced periodontal tissue regeneration by periodontal cell implantation. J. Clin. Periodontol..

[31] Kobolak J., Dinnyes A., Memic A., Khademhosseini A., Mobasheri A. (2016). Mesenchymal stem cells: Identification, phenotypic characterization, biological properties and potential for regenerative medicine through biomaterial micro-engineering of their niche. Methods.

[32] Huang G.-J., Gronthos S., Shi S. (2009). Mesenchymal stem cells derived from dental tissues vs. those from other sources: Their biology and role in regenerative medicine. J. Dent. Res..

[33] Gronthos S., Mankani M., Brahim J., Robey P.G., Shi S. (2000). Postnatal human dental pulp stem cells (DPSCs) in vitro and in vivo. Proc. Natl. Acad. Sci. USA.

[34] Miura M., Gronthos S., Zhao M., Lu B., Fisher L.W., Robey P.G., Shi S. (2003). SHED: Stem cells from human exfoliated deciduous teeth. Proc. Natl. Acad. Sci. USA.

[35] Seo B.-M., Miura M., Gronthos S., Bartold P.M., Batouli S., Brahim J., Young M., Robey P.G., Wang C.Y., Shi S. (2004). Investigation of multipotent postnatal stem cells from human periodontal ligament. Lancet.

[36] Morsczeck C., Moehl C., Götz W., Heredia A., Schäffer T., Eckstein N., Sippel C., Hoffmann K. (2005). In vitro differentiation of human dental follicle cells with dexamethasone and insulin. Cell Biol. Int..

[37] Sonoyama W., Liu Y., Yamaza T., Tuan R.S., Wang S., Shi S., Huang G.T.-J. (2008). Characterization of the apical papilla and its residing stem cells from human immature permanent teeth: A pilot study. J. Endod..

[38] Zhao L., Burguera E.F., Xu H.H., Amin N., Ryou H., Arola D.D. (2010). Fatigue and human umbilical cord stem cell seeding characteristics of calcium phosphate–chitosan–biodegradable fiber scaffolds. Biomaterials.

[39] Can A., Karahuseyinoglu S. (2007). Concise review: Human umbilical cord stroma with regard to the source of fetus-derived stem cells. Stem Cells.

[40] Baethge C., Goldbeck-Wood S., Mertens S. (2019). SANRA—A scale for the quality assessment of narrative review articles. Res. Integr. Peer Rev..

[41] Darveau R.P. (2010). Periodontitis: A polymicrobial disruption of host homeostasis. Nat. Rev. Microbiol..

[42] Lourenço T.G.B., Heller D., Silva-Boghossian C.M., Cotton S.L., Paster B.J., Colombo A.P.V. (2014). Microbial signature profiles of periodontally healthy and diseased patients. J. Clin. Periodontol..

[43] Perez-Chaparro P., Gonçalves C., Figueiredo L., Faveri M., Lobão E., Tamashiro N., Duarte P., Feres M. (2014). Newly identified pathogens associated with periodontitis: A systematic review. J. Dent. Res..

[44] Pérez-Chaparro P.J., Duarte P.M., Shibli J.A., Montenegro S., Lacerda Heluy S., Figueiredo L.C., Faveri M., Feres M. (2016). The current weight of evidence of the microbiologic profile associated with peri-implantitis: A systematic review. J. Periodontol..

[45] Feres M., Teles F., Teles R., Figueiredo L.C., Faveri M. (2016). The subgingival periodontal microbiota of the aging mouth. Periodontol. 2000.

[46] Haubek D., Ennibi O.-K., Poulsen K., Væth M., Poulsen S., Kilian M. (2008). Risk of aggressive periodontitis in adolescent carriers of the JP2 clone of Aggregatibacter (Actinobacillus) actinomycetemcomitans in Morocco: A prospective longitudinal cohort study. Lancet.

[47] Amaliya A., Laine M.L., Delanghe J.R., Loos B.G., Van Wijk A.J., Van der Velden U. (2015). Java project on periodontal diseases: Periodontal bone loss in relation to environmental and systemic conditions. J Clin. Periodontol..

[48] Mombelli A., Casagni F., Madianos P.N. (2002). Can presence or absence of periodontal pathogens distinguish between subjects with chronic and aggressive periodontitis? A systematic review. J. Clin. Periodontol..

[49] Pillet S., Pozzetto B., Roblin X. (2016). Cytomegalovirus and ulcerative colitis: Place of antiviral therapy. World J. Gastroenterol..

[50] Slots J. (2015). Periodontal herpesviruses: Prevalence, pathogenicity, systemic risk. Periodontol. 2000.

[51] Yong-wei F., Yong-qing G., Hong-zhi X. (2014). Valacyclovir as an adjunct to full-mouth scaling and root planing of advanced chronic periodontitis: A randomized clinical trail. Shanghai J. Stomatol..

[52] Sunde P.T., Olsen I., Enersen M., Grinde B. (2008). Patient with severe periodontitis and subgingival Epstein-Barr virus treated with antiviral therapy. J. Clin. Virol..

[53] Mäntylä P., Stenman M., Kinane D.F., Tikanoja S., Luoto H., Salo T., Sorsa T. (2003). Gingival crevicular fluid collagenase-2 (MMP-8) test stick for chair-side monitoring of periodontitis. J. Periodontal Res..

[54] Kinane D.F., Demuth D.R., Gorr S.U., Hajishengallis G.N., Martin M.H. (2007). Human variability in innate immunity. Periodontol. 2000.

[55] Hajishengallis G., Lamont R.J. (2012). Beyond the red complex and into more complexity: The polymicrobial synergy and dysbiosis (PSD) model of periodontal disease etiology. Mol. Oral Microbiol..

[56] Kinane D.F., Hajishengallis G. (2009). Polymicrobial infections, biofilms, and beyond. J. Clin. Periodontol..

[57] Benakanakere M., Kinane D.F. (2012). Innate cellular responses to the periodontal biofilm. Front Oral Biol..

[58] Graves D. (2008). Cytokines that promote periodontal tissue destruction. J. Periodontol..

[59] Pan W., Wang Q., Chen Q. (2019). The cytokine network involved in the host immune response to periodontitis. Int. J. Oral Sci..

[60] Gemmell E., Marshall R.I., Seymour G.J. (1997). Cytokines and prostaglandins in immune homeostasis and tissue destruction in periodontal disease. Periodontol. 2000.

[61] Sorsa T., Gursoy U.K., Nwhator S., Hernandez M., Tervahartiala T., Leppilahti J., Gursoy M., Könönen E., Emingil G., Pussinen P.J. (2016). Analysis of matrix metalloproteinases, especially MMP-8, in gingival crevicular fluid, mouthrinse and saliva for monitoring periodontal diseases. Periodontol. 2000.

[62] Gemmell E., Seymour G.J. (2004). Immunoregulatory control of Th1/Th2 cytokine profiles in periodontal disease. Periodontol. 2000.

[63] Aranha A.M.F., Repeke C.E., Garlet T.P., Vieira A.E., Campanelli A.P., Trombone A.P.F., Letra A., Silva R.M., Garlet G.P. (2013). Evidence supporting a protective role for th9 and th22 cytokines in human and experimental periapical lesions. J. Endod..

[64] Eskan M.A., Jotwani R., Abe T., Chmelar J., Lim J.-H., Liang S., Ciero P.A., Krauss J.L., Li F., Rauner M. (2012). The leukocyte integrin antagonist Del-1 inhibits IL-17-mediated inflammatory bone loss. Nat. Immunol..

[65] Figueredo C., Lira-Junior R., Love R. (2019). T and B cells in periodontal disease: New functions in a complex scenario. Int. J. Mol. Sci..

[66] Abbas F., Van der Velden U., Hart A., Moorer W., Vroom T.M., Scholte G. (1986). Bleeding/plaque ratio and the development of gingival inflammation. J. Clin. Periodontol..

[67] Winkel E., Abbas F., Van der Velden U., Vroom T.M., Scholte G., Hart A. (1987). Experimental gingivitis in relation to age in individuals not susceptible to periodontal destruction. J. Clin. Periodontol..

[68] Kinane D., Attström R. (2005). Advances in the pathogenesis of periodontitis. Group B consensus report of the fifth European Workshop in Periodontology. J. Clin. Periodontol..

[69] Joss A., Adler R., Lang N.P. (1994). Bleeding on probing. A parameter for monitoring periodontal conditions in clinical practice. J. Clin. Periodontol..

[70] Lang N.P., Adler R., Joss A., Nyman S. (1990). Absence of bleeding on probing an indicator of periodontal stability. J. Clin. Periodontol..

[71] Löe H., Theilade E., Jensen S.B. (1965). Experimental gingivitis in man. J. Periodontol..

[72] Theilade E., Wright W., Jensen S.B., Löe H. (1966). Experimental gingivitis in man: II. A longitudinal clinical and bacteriological investigation. J. Periodontal Res..

[73] Trombelli L., Scapoli C., Tatakis D.N., Grassi L. (2005). Modulation of clinical expression of plaque-induced gingivitis: Effects of personality traits, social support and stress. J. Clin. Periodontol..

[74] Trombelli L., Tatakis D.N., Scapoli C., Bottega S., Orlandini E., Tosi M. (2004). Modulation of clinical expression of plaque-induced gingivitis: II. Identification of “high-responder” and “low-responder” subjects. J. Clin. Periodontol..

[75] Tatakis D.N., Trombelli L. (2004). Modulation of clinical expression of plaque-induced gingivitis: I. Background review and rationale. J. Clin. Periodontol..

[76] Scapoli C., Mamolini E., Trombelli L. (2007). Role of IL-6, TNF-A and LT-A variants in the modulation of the clinical expression of plaque-induced gingivitis. J. Clin. Periodontol..

[77] Trombelli L., Farina R., Minenna L., Carrieri A., Scapoli C., Tatakis D.N. (2008). Experimental gingivitis: Reproducibility of plaque accumulation and gingival inflammation parameters in selected populations during a repeat trial. J. Clin. Periodontol..

[78] Trombelli L., Scapoli C., Carrieri A., Giovannini G., Calura G., Farina R. (2010). Interleukin-1β levels in gingival crevicular fluid and serum under naturally occurring and experimentally induced gingivitis. J. Clin. Periodontol..

[79] Offenbaceer S., Odle B., Van Dyke T. (1986). The use of crevicular fluid prostaglandin E2 levels as a predictor of periodontal attachment loss. J. Periodontal Res..

[80] Stashenko P. (1990). The role of immune cytokines in the pathogenesis of periapical lesions. Dent. Traumatol..

[81] Wilton J., Griffiths G., Curtis M., Maiden M., Gillett I., Wilson D., Sterne J., Johnson N. (1988). Detection of high-risk groups and individuals for periodontal diseases: Systemic predisposition and markers of general health. J. Clin. Periodontol..

[82] Ellis J.S., Seymour R.A., Steele J.G., Robertson P., Butler T.J., Thomason J.M. (1999). Prevalence of gingival overgrowth induced by calcium channel blockers: A community-based study. J. Periodontol..

[83] Wu M., Chen S.-W., Jiang S.-Y. (2015). Relationship between gingival inflammation and pregnancy. Mediators Inflamm..

[84] Barr C., Lopez M., Rua-Dobles A. (1992). Periodontal changes by HIV serostatus in a cohort of homosexual and bisexual men. J. Clin. Periodontol..

[85] Kinane D.F., Lappin D.F. (2002). Immune processes in periodontal disease: A review. Ann. Periodontol..

[86] Van der Velden U., Abbas F., Armand S., Loos B., Timmerman M., Van der Weijden G., Van Winkelhoff A., Winkel E. (2006). Java project on periodontal diseases. The natural development of periodontitis: Risk factors, risk predictors and risk determinants. J. Clin. Periodontol..

[87] Shapira L., Wilensky A., Kinane D.F. (2005). Effect of genetic variability on the inflammatory response to periodontal infection. J. Clin. Periodontol..

[88] Kinane D.F., Shiba H., Hart T.C. (2005). The genetic basis of periodontitis. Periodontol. 2000.

[89] Baylin S.B. (2005). DNA methylation and gene silencing in cancer. Nat. Clin. Pract. Oncol..

[90] Benakanakere M., Abdolhosseini M., Hosur K., Finoti L., Kinane D. (2015). TLR2 promoter hypermethylation creates innate immune dysbiosis. J. Dent. Res..

[91] De Jong T., Bakker A., Everts V., Smit T. (2017). The intricate anatomy of the periodontal ligament and its development: Lessons for periodontal regeneration. J. Periodontal Res..

[92] Bartold P.M., McCulloch C.A. (2013). Information generation and processing systems that regulate periodontal structure and function. Periodontol. 2000.

[93] Park C.H., Kim K.-H., Lee Y.-M., Seol Y.-J. (2016). Advanced engineering strategies for periodontal complex regeneration. Materials.

[94] Lin J.D., Jang A.T., Kurylo M.P., Hurng J., Yang F., Yang L., Pal A., Chen L., Ho S.P. (2017). Periodontal ligament entheses and their adaptive role in the context of dentoalveolar joint function. Dent. Mater..

[95] Lee J.H., Pryce B.A., Schweitzer R., Ryder M.I., Ho S.P. (2015). Differentiating zones at periodontal ligament–bone and periodontal ligament–cementum entheses. J. Periodontal Res..

[96] Park C.H., Rios H.F., Jin Q., Sugai J.V., Padial-Molina M., Taut A.D., Flanagan C.L., Hollister S.J., Giannobile W.V. (2012). Tissue engineering bone-ligament complexes using fiber-guiding scaffolds. Biomaterials.

[97] Lee J.-H., Lin J.D., Fong J.I., Ryder M.I., Ho S.P. (2013). The adaptive nature of the bone-periodontal ligament-cementum complex in a ligature-induced periodontitis rat model. BioMed Res. Int..

[98] Silva N., Abusleme L., Bravo D., Dutzan N., Garcia-Sesnich J., Vernal R., Hernandez M., Gamonal J. (2015). Host response mechanisms in periodontal diseases. J. Appl. Oral Sci..

[99] Cho Y.-D., Kim K.-H., Lee Y.-M., Ku Y., Seol Y.-J. (2021). Periodontal wound healing and tissue regeneration: A narrative review. Pharmaceuticals.

[100] Foster B.L., Nagatomo K.J., Nociti Jr F.H., Fong H., Dunn D., Tran A.B., Wang W., Narisawa S., Millán J.L., Somerman M.J. (2012). Central role of pyrophosphate in acellular cementum formation. PLoS ONE.

[101] Matalová E., Lungová V., Sharpe P. (2015). Development of tooth and associated structures. Stem Cell Biology and Tissue Engineering in Dental Sciences.

[102] Foster B.L., Popowics T.E., Fong H.K., Somerman M.J. (2007). Advances in defining regulators of cementum development and periodontal regeneration. Curr. Top. Dev. Biol..

[103] Arzate H., Zeichner-David M., Mercado-Celis G. (2015). Cementum proteins: Role in cementogenesis, biomineralization, periodontium formation and regeneration. Periodontol. 2000.

[104] Hoz L., Romo E., Zeichner-David M., Sanz M., Nuñez J., Gaitán L., Mercado G., Arzate H. (2012). Cementum protein 1 (CEMP1) induces differentiation by human periodontal ligament cells under three-dimensional culture conditions. Cell Biol. Int..

[105] Bosshardt D.D., Schroeder H.E. (1996). Cementogenesis reviewed: A comparison between human premolars and rodent molars. Anat. Rec..

[106] Bar-Kana I., Savion N., Narayanan A., Pitaru S. (1998). Cementum attachment protein manifestation is restricted to the mineralized tissue forming cells of the periodontium. Eur. J. Oral Sci..

[107] Zhu W., Liang M. (2015). Periodontal ligament stem cells: Current status, concerns, and future prospects. Stem Cells Int..

[108] Lemaitre M., Monsarrat P., Blasco-Baque V., Loubières P., Burcelin R., Casteilla L., Planat-Bénard V., Kémoun P. (2017). Periodontal tissue regeneration using syngeneic adipose-derived stromal cells in a mouse model. Stem Cells Transl. Med..

[109] Yang B., Chen G., Li J., Zou Q., Xie D., Chen Y., Wang H., Zheng X., Long J., Tang W. (2012). Tooth root regeneration using dental follicle cell sheets in combination with a dentin matrix-based scaffold. Biomaterials.

[110] Crossman J., Elyasi M., El-Bialy T., Mir C.F. (2018). Cementum regeneration using stem cells in the dog model: A systematic review. Arch. Oral Biol..

[111] Yang Z.H., Zhang X.J., Dang N.N., Ma Z.F., Xu L., Wu J.J., Sun Y.J., Duan Y.Z., Lin Z., Jin Y. (2009). Apical tooth germ cell-conditioned medium enhances the differentiation of periodontal ligament stem cells into cementum/periodontal ligament-like tissues. J. Periodontal Res..

[112] Owaki T., Shimizu T., Yamato M., Okano T. (2014). Cell sheet engineering for regenerative medicine: Current challenges and strategies. Biotechnol. J..

[113] Pedroni A.C.F., Sarra G., de Oliveira N.K., Moreira M.S., Deboni M.C.Z., Marques M.M. (2019). Cell sheets of human dental pulp stem cells for future application in bone replacement. Clin. Oral Investig..

[114] Yorukoglu A.C., Kiter A., Akkaya S., Satiroglu-Tufan N.L., Tufan A.C. (2017). A concise review on the use of mesenchymal stem cells in cell sheet-based tissue engineering with special emphasis on bone tissue regeneration. Stem Cells Int..

[115] Iwata T., Yamato M., Tsuchioka H., Takagi R., Mukobata S., Washio K., Okano T., Ishikawa I. (2009). Periodontal regeneration with multi-layered periodontal ligament-derived cell sheets in a canine model. Biomaterials.

[116] Iwata T., Washio K., Yoshida T., Ishikawa I., Ando T., Yamato M., Okano T. (2015). Cell sheet engineering and its application for periodontal regeneration. J. Tissue Eng. Reg. Med..

[117] Mao L., Liu J., Zhao J., Chang J., Xia L., Jiang L., Wang X., Lin K., Fang B. (2015). Effect of micro-nano-hybrid structured hydroxyapatite bioceramics on osteogenic and cementogenic differentiation of human periodontal ligament stem cell via Wnt signaling pathway. Int. J. Nanomedicine..

[118] Zhu W., Zhang Q., Zhang Y., Cen L., Wang J. (2015). PDL regeneration via cell homing in delayed replantation of avulsed teeth. J. Transl. Med..

[119] Bartold P.M., Shi S., Gronthos S. (2006). Stem cells and periodontal regeneration. Periodontol. 2000.

[120] Catón J., Bostanci N., Remboutsika E., De Bari C., Mitsiadis T.A. (2011). Future dentistry: Cell therapy meets tooth and periodontal repair and regeneration. J. Cell. Mol. Med..

[121] Mitsiadis T.A., Woloszyk A., Jiménez-Rojo L. (2012). Nanodentistry: Combining nanostructured materials and stem cells for dental tissue regeneration. Nanomedicine.

[122] Shi H., Zong W., Xu X., Chen J. (2018). Improved biphasic calcium phosphate combined with periodontal ligament stem cells may serve as a promising method for periodontal regeneration. Am. J. Transl. Res..

[123] Xu X.Y., Li X., Wang J., He X.T., Sun H.H., Chen F.M. (2019). Concise review: Periodontal tissue regeneration using stem cells: Strategies and translational considerations. Stem Cells Transl. Med..

[124] Mohammed E., Khalil E., Sabry D. (2018). Effect of adipose-derived stem cells and their exo as adjunctive therapy to nonsurgical periodontal treatment: A histologic and histomorphometric study in rats. Biomolecules.

[125] Duan X., Tu Q., Zhang J., Ye J., Sommer C., Mostoslavsky G., Kaplan D., Yang P., Chen J. (2011). Application of induced pluripotent stem (iPS) cells in periodontal tissue regeneration. J. Cell. Physiol..

[126] Bosshardt D.D., Sculean A. (2009). Does periodontal tissue regeneration really work?. Periodontol. 2000.

[127] Siaili M., Chatzopoulou D., Gillam D. (2018). An overview of periodontal regenerative procedures for the general dental practitioner. Saudi Dent. J..

[128] Bottino M.C., Thomas V., Schmidt G., Vohra Y.K., Chu T.-M.G., Kowolik M.J., Janowski G.M. (2012). Recent advances in the development of GTR/GBR membranes for periodontal regeneration—A materials perspective. Dent. Mater..

[129] Almutairi A.S. (2018). Case Report: Managing the postoperative exposure of a non-resorbable membrane surgically. F1000Res.

[130] Pretzl B., Kim T.S., Holle R., Eickholz P. (2008). Long-term results of guided tissue regeneration therapy with non-resorbable and bioabsorbable barriers. IV. A case series of infrabony defects after 10 years. J. Periodontol..

[131] Corinaldesi G., Lizio G., Badiali G., Morselli-Labate A.M., Marchetti C. (2011). Treatment of intrabony defects after impacted mandibular third molar removal with bioabsorbable and non-resorbable membranes. J. Periodontol..

[132] Moshaverinia A., Chen C., Xu X., Akiyama K., Ansari S., Zadeh H.H., Shi S. (2014). Bone regeneration potential of stem cells derived from periodontal ligament or gingival tissue sources encapsulated in RGD-modified alginate scaffold. Tissue Eng. Part A.

[133] Bollati D., Morra M., Cassinelli C., Cascardo G. (2018). Implantable Devices Having Antibacterial Properties and Multifunctional Surfaces. U.S. Patent.

[134] Nyamsuren E., Bayarchimeg B., Urjinlkham J., Oyun-enkh P., Kh O., Batsuuri M., Lu S.-l. (2018). Efficacy of natural biopolymer chitosan membrane for guided tissue regeneration. Innovation.

[135] Cui J., Jiang B., Liang J., Sun C., Lan J., Sun X., Huang H., Sun K., Xu X. (2011). Preparation and characterization of chitosan/β-GP membranes for guided bone regeneration. J. Wuhan Univ. Technol. Mater. Sci. Ed..

[136] Xue Y., Hong X., Gao J., Shen R., Ye Z. (2019). Preparation and biological characterization of the mixture of poly (lactic-co-glycolic acid)/chitosan/Ag nanoparticles for periodontal tissue engineering. Int. J. Nanomedicine.

[137] Lausch A.J., Chong L.C., Uludag H., Sone E.D. (2018). Multiphasic collagen scaffolds for engineered tissue interfaces. Adv. Funct. Mater..

[138] Zhou T., Liu X., Sui B., Liu C., Mo X., Sun J. (2017). Development of fish collagen/bioactive glass/chitosan composite nanofibers as a GTR/GBR membrane for inducing periodontal tissue regeneration. Biomed. Mater..

[139] Mahajan A., Kedige S. (2015). Periodontal bone regeneration in intrabony defects using osteoconductive bone graft versus combination of osteoconductive and osteostimulative bone graft: A comparative study. Dent. Res. J..

[140] Zhou M., Geng Y.-m., Li S.-y., Yang X.-b., Che Y.-j., Pathak J.L., Wu G. (2019). Nanocrystalline hydroxyapatite-based scaffold adsorbs and gives sustained release of osteoinductive growth factor and facilitates bone regeneration in mice ectopic model. J. Nanomater..

[141] Chen M., Xu Y., Zhang T., Ma Y., Liu J., Yuan B., Chen X., Zhou P., Zhao X., Pang F. (2019). Mesenchymal stem cell sheets: A new cell-based strategy for bone repair and regeneration. Biotechnol. Lett..

[142] Ivanovski S., Vaquette C., Gronthos S., Hutmacher D., Bartold P. (2014). Multiphasic scaffolds for periodontal tissue engineering. J. Dent. Res..

[143] Sakkas A., Wilde F., Heufelder M., Winter K., Schramm A. (2017). Autogenous bone grafts in oral implantology—is it still a “gold standard”? A consecutive review of 279 patients with 456 clinical procedures. Int. J. Implant Dent..

[144] Reynolds M.A., Aichelmann-Reidy M.E., Branch-Mays G.L. (2010). Regeneration of periodontal tissue: Bone replacement grafts. Dent. Clin. N. Am..

[145] Keith Jr J.D., Petrungaro P., Leonetti J.A., Elwell Jr C.W., Zeren K.J., Caputo C., Nikitakis N.G., Schöpf C., Warner M.M. (2006). Clinical and histologic evaluation of a mineralized block allograft: Results from the developmental period (2001-2004). Int. J. Periodontics Restorative Dent..

[146] Piattelli A., Scarano A., Corigliano M., Piattelli M. (1996). Comparison of bone regeneration with the use of mineralized and demineralized freeze-dried bone allografts: A histological and histochemical study in man. Biomaterials.

[147] Liu X., Li Q., Wang F., Wang Z. (2016). Maxillary sinus floor augmentation and dental implant placement using dentin matrix protein-1 gene-modified bone marrow stromal cells mixed with deproteinized boving bone: A comparative study in beagles. Arch. Oral Biol..

[148] Sheikh Z., Hamdan N., Ikeda Y., Grynpas M., Ganss B., Glogauer M. (2017). Natural graft tissues and synthetic biomaterials for periodontal and alveolar bone reconstructive applications: A review. Biomater. Res..

[149] Zhang Y., Xu H.H., Takagi S., Chow L.C. (2006). In-situ hardening hydroxyapatite-based scaffold for bone repair. J. Mater. Sci. Mater. Med..

[150] Belal M.H., Al-Noamany F.A., El-Tonsy M.M., El-Guindy H.M., Ishikawa I. (2005). Treatment of human class II furcation defects using connective tissue grafts, bioabsorbable membrane, and resorbable hydroxylapatite: A comparative study. J. Int. Acad. Periodontol..

[151] Zhang Y., Xu H.H. (2005). Effects of synergistic reinforcement and absorbable fiber strength on hydroxyapatite bone cement. J. Biomed. Mater. Res. A.

[152] Hayashi C., Kinoshita A., Oda S., Mizutani K., Shirakata Y., Ishikawa I. (2006). Injectable calcium phosphate bone cement provides favorable space and a scaffold for periodontal regeneration in dogs. J. Periodontol..

[153] Chu K., Oshida Y., Hancock E., Kowolik M.J., Barco T., Zunt S. (2004). Hydroxyapatite/PMMA composites as bone cements. Biomed. Mater. Eng..

[154] Fu Q., Saiz E., Rahaman M.N., Tomsia A.P. (2013). Tissue Engineering: Toward Strong and Tough Glass and Ceramic Scaffolds for Bone Repair (Adv. Funct. Mater. 44/2013). Adv. Funct. Mater..

[155] Tsai H.-C., Li Y.-C., Young T.-H., Chen M.-H. (2016). Novel microinjector for carrying bone substitutes for bone regeneration in periodontal diseases. J. Formos. Med. Assoc..

[156] Simon Jr C.G., Khatri C.A., Wight S.A., Wang F.W. (2002). Preliminary report on the biocompatibility of a moldable, resorbable, composite bone graft consisting of calcium phosphate cement and poly (lactide-co-glycolide) microspheres. J. Orthop. Res..

[157] Brown W.E., Chow L.C. (1986). Combinations of Sparingly Soluble Calcium Phosphates in Slurries and Pastes as Mineralizers and Cements. U.S. Patent.

[158] Takagi S., Chow L.C., Hirayama S., Eichmiller F.C. (2003). Properties of elastomeric calcium phosphate cement–chitosan composites. Dent. Mater..

[159] Xu H.H., Wang P., Wang L., Bao C., Chen Q., Weir M.D., Chow L.C., Zhao L., Zhou X., Reynolds M.A. (2017). Calcium phosphate cements for bone engineering and their biological properties. Bone Res..

[160] Villalona G.A., Udelsman B., Duncan D.R., McGillicuddy E., Sawh-Martinez R.F., Hibino N., Painter C., Mirensky T., Erickson B., Shinoka T. (2010). Cell-seeding techniques in vascular tissue engineering. Tissue Eng. Part B Rev..

[161] Wang P., Song Y., Weir M.D., Sun J., Zhao L., Simon C.G., Xu H.H. (2016). A self-setting iPSMSC-alginate-calcium phosphate paste for bone tissue engineering. Dent. Mater..

[162] Grosfeld E.-C., Hoekstra J.W.M., Herber R.-P., Ulrich D.J., Jansen J.A., van den Beucken J.J. (2016). Long-term biological performance of injectable and degradable calcium phosphate cement. Biomed. Mater..

[163] Song Y., Zhang C., Wang P., Wang L., Bao C., Weir M.D., Reynolds M.A., Ren K., Zhao L., Xu H.H. (2017). Engineering bone regeneration with novel cell-laden hydrogel microfiber-injectable calcium phosphate scaffold. Mater. Sci. Eng. C Mater. Biol. Appl..

[164] Zhao L., Weir M.D., Xu H.H. (2010). An injectable calcium phosphate-alginate hydrogel-umbilical cord mesenchymal stem cell paste for bone tissue engineering. Biomaterials.

[165] Mitsiadis T., Feki A., Papaccio G., Catón J. (2011). Dental pulp stem cells, niches, and notch signaling in tooth injury. Adv. Dent. Res..

[166] Wang P., Liu X., Zhao L., Weir M.D., Sun J., Chen W., Man Y., Xu H.H. (2015). Bone tissue engineering via human induced pluripotent, umbilical cord and bone marrow mesenchymal stem cells in rat cranium. Acta Biomater..

[167] Wang L., Zhang C., Li C., Weir M.D., Wang P., Reynolds M.A., Zhao L., Xu H.H. (2016). Injectable calcium phosphate with hydrogel fibers encapsulating induced pluripotent, dental pulp and bone marrow stem cells for bone repair. Mater. Sci. Eng. C Mater. Biol. Appl..

[168] Schweikle M., Zinn T., Lund R., Tiainen H. (2018). Injectable synthetic hydrogel for bone regeneration: Physicochemical characterisation of a high and a low pH gelling system. Mater. Sci. Eng. C Mater. Biol. Appl..

[169] Zhang C., Hu K., Liu X., Reynolds M.A., Bao C., Wang P., Zhao L., Xu H.H. (2017). Novel hiPSC-based tri-culture for pre-vascularization of calcium phosphate scaffold to enhance bone and vessel formation. Mater. Sci. Eng. C Mater. Biol. Appl..

[170] Batool F., Strub M., Petit C., Bugueno I.M., Bornert F., Clauss F., Huck O., Kuchler-Bopp S., Benkirane-Jessel N. (2018). Periodontal tissues, maxillary jaw bone, and tooth regeneration approaches: From animal models analyses to clinical applications. Nanomaterials.

[171] Besinis A., De Peralta T., Tredwin C.J., Handy R.D. (2015). Review of nanomaterials in dentistry: Interactions with the oral microenvironment, clinical applications, hazards, and benefits. ACS Nano.

[172] Polini A., Bai H., Tomsia A.P. (2013). Dental applications of nanostructured bioactive glass and its composites. Wiley Interdiscip. Rev. Nanomed. Nanobiotechnol..

[173] Li G., Zhou T., Lin S., Shi S., Lin Y. (2017). Nanomaterials for craniofacial and dental tissue engineering. J. Dent. Res..

[174] Li J., Li Y., Ma S., Gao Y., Zuo Y., Hu J. (2010). Enhancement of bone formation by BMP-7 transduced MSCs on biomimetic nano-hydroxyapatite/polyamide composite scaffolds in repair of mandibular defects. J. Biomed. Mater. Res. Part A.

[175] Xia Y., Chen H., Zhang F., Bao C., Weir M.D., Reynolds M.A., Ma J., Gu N., Xu H.H. (2018). Gold nanoparticles in injectable calcium phosphate cement enhance osteogenic differentiation of human dental pulp stem cells. Nanomed. Nanotechnol. Biol. Med..

[176] Yang J.W., Shin Y.Y., Seo Y., Kim H.-S. (2020). Therapeutic functions of stem cells from oral cavity: An update. Int. J. Mol. Sci..

[177] Sybil D., Jain V., Mohanty S., Husain S.A. (2020). Oral stem cells in intraoral bone formation. J. Oral Biosci..

[178] Gronthos S., Brahim J., Li W., Fisher L., Cherman N., Boyde A., DenBesten P., Robey P.G., Shi S. (2002). Stem cell properties of human dental pulp stem cells. J. Dent. Res..

[179] Spagnuolo G., Codispoti B., Marrelli M., Rengo C., Rengo S., Tatullo M. (2018). Commitment of oral-derived stem cells in dental and maxillofacial applications. Dent. J..

[180] Capparè P., Tetè G., Sberna M.T., Panina-Bordignon P. (2020). The emerging role of stem cells in regenerative dentistry. Curr. Gene Ther..

[181] Wang D., Wang Y., Tian W., Pan J. (2019). Advances of tooth-derived stem cells in neural diseases treatments and nerve tissue regeneration. Cell Prolif..

[182] Tziafas D., Kodonas K. (2010). Differentiation potential of dental papilla, dental pulp, and apical papilla progenitor cells. J. Endod..

[183] Graziano A., d’Aquino R., Angelis M.G.C.D., De Francesco F., Giordano A., Laino G., Piattelli A., Traini T., De Rosa A., Papaccio G. (2008). Scaffold’s surface geometry significantly affects human stem cell bone tissue engineering. J. Cell. Physiol..

[184] Yu J., He H., Tang C., Zhang G., Li Y., Wang R., Shi J., Jin Y. (2010). Differentiation potential of STRO-1+ dental pulp stem cells changes during cell passaging. BMC Cell Biol..

[185] Papaccio G., Graziano A., d’Aquino R., Graziano M.F., Pirozzi G., Menditti D., De Rosa A., Carinci F., Laino G. (2006). Long-term cryopreservation of dental pulp stem cells (SBP-DPSCs) and their differentiated osteoblasts: A cell source for tissue repair. J. Cell. Physiol..

[186] Trubiani O., Pizzicannella J., Caputi S., Marchisio M., Mazzon E., Paganelli R., Paganelli A., Diomede F. (2019). Periodontal ligament stem cells: Current knowledge and future perspectives. Stem Cells Dev..

[187] Liu L., Michowski W., Kolodziejczyk A., Sicinski P. (2019). The cell cycle in stem cell proliferation, pluripotency and differentiation. Nat. Cell Biol..

[188] Su W.-T., Chiou W.-L., Yu H.-H., Huang T.-Y. (2016). Differentiation potential of SHEDs using biomimetic periosteum containing dexamethasone. Mater. Sci. Eng. C Mater. Biol. Appl..

[189] Gao X., Shen Z., Guan M., Huang Q., Chen L., Qin W., Ge X., Chen H., Xiao Y., Lin Z. (2018). Immunomodulatory role of stem cells from human exfoliated deciduous teeth on periodontal regeneration. Tissue Eng. Part A.

[190] Nakamura S., Yamada Y., Katagiri W., Sugito T., Ito K., Ueda M. (2009). Stem cell proliferation pathways comparison between human exfoliated deciduous teeth and dental pulp stem cells by gene expression profile from promising dental pulp. J. Endod..

[191] Chrepa V., Pitcher B., Henry M.A., Diogenes A. (2017). Survival of the apical papilla and its resident stem cells in a case of advanced pulpal necrosis and apical periodontitis. J. Endod..

[192] Nada O.A., El Backly R.M. (2018). Stem cells from the apical papilla (SCAP) as a tool for endogenous tissue regeneration. Front. Bioeng. Biotechnol..

[193] Hu B., Nadiri A., Kuchler-Bopp S., Perrin-Schmitt F., Peters H., Lesot H. (2006). Tissue engineering of tooth crown, root, and periodontium. Tissue Eng..

[194] Hu B., Unda F., Bopp-Kuchler S., Jimenez L., Wang X., Haikel Y., Wang S., Lesot H. (2006). Bone marrow cells can give rise to ameloblast-like cells. J. Dent. Res..

[195] Hasegawa N., Kawaguchi H., Hirachi A., Takeda K., Mizuno N., Nishimura M., Koike C., Tsuji K., Iba H., Kato Y. (2006). Behavior of transplanted bone marrow–derived mesenchymal stem cells in periodontal defects. J. Periodontol..

[196] Yang Y., Rossi F.M., Putnins E.E. (2010). Periodontal regeneration using engineered bone marrow mesenchymal stromal cells. Biomaterials.

[197] Ohazama A., Modino S., Miletich I., Sharpe P. (2004). Stem-cell-based tissue engineering of murine teeth. J. Dent. Res..

[198] Evans M.J., Kaufman M.H. (1981). Establishment in culture of pluripotential cells from mouse embryos. Nature.

[199] Inanç B., Elçin A.E., Elçin Y.M. (2009). In vitro differentiation and attachment of human embryonic stem cells on periodontal tooth root surfaces. Tissue Eng. Part A.

[200] Ning F., Guo Y., Tang J., Zhou J., Zhang H., Lu W., Gao Y., Wang L., Pei D., Duan Y. (2010). Differentiation of mouse embryonic stem cells into dental epithelial-like cells induced by ameloblasts serum-free conditioned medium. Biochem. Biophys. Res. Commun..

[201] Takahashi K., Yamanaka S. (2006). Induction of pluripotent stem cells from mouse embryonic and adult fibroblast cultures by defined factors. Cell.

[202] Wada N., Wang B., Lin N.H., Laslett A.L., Gronthos S., Bartold P.M. (2011). Induced pluripotent stem cell lines derived from human gingival fibroblasts and periodontal ligament fibroblasts. J. Periodontal Res..

[203] Yan X., Qin H., Qu C., Tuan R.S., Shi S., Huang G.T.-J. (2010). iPS cells reprogrammed from human mesenchymal-like stem/progenitor cells of dental tissue origin. Stem Cells Dev..

[204] Andrukhov O., Behm C., Blufstein A., Rausch-Fan X. (2019). Immunomodulatory properties of dental tissue-derived mesenchymal stem cells: Implication in disease and tissue regeneration. World J. Stem Cells.

[205] Alatyyat S.M., Alasmari H.M., Aleid O.A., Abdel-Maksoud M.S., Elsherbiny N. (2020). Umbilical cord stem cells: Background, processing and applications. Tissue Cell.

[206] Szepesi Á., Matula Z., Szigeti A., Várady G., Szalma J., Szabó G., Uher F., Sarkadi B., Német K. (2016). In vitro characterization of human mesenchymal stem cells isolated from different tissues with a potential to promote complex bone regeneration. Stem Cells Int..

[207] Eliane G., Agnes D., Helene E., Dominique T., Gerard S., Pierre L., Scott C., Denis E., Joanne K., Judith B. (2011). Hematopoietic reconstitution in a patient with Fanconi’s anemia by means of umbilical-cord blood from an HLA-identical sibling (1989). Cell. Ther. Transplant..

[208] Gluckman E. (2001). Hematopoietic stem-cell transplants using umbilical-cord blood. Mass Med. Soc..

[209] Revencu T., Trifan V., Nacu L., Gutium T., Globa L., Motoc A., Nacu V. (2013). Collection, isolation and characterization of the stem cells of umbilical cord blood. Rom. J. Morphol. Embryol..

[210] Matsumoto T., Mugishima H. (2009). Non-hematopoietic stem cells in umbilical cord blood. Int. J Stem Cells.

[211] Cardoso T.C., Ferrari H.F., Garcia A.F., Novais J.B., Silva-Frade C., Ferrarezi M.C., Andrade A.L., Gameiro R. (2012). Isolation and characterization of Wharton’s jelly-derived multipotent mesenchymal stromal cells obtained from bovine umbilical cord and maintained in a defined serum-free three-dimensional system. BMC Biotechnol..

[212] Saleh R., Reza H.M. (2017). Short review on human umbilical cord lining epithelial cells and their potential clinical applications. Stem Cell Res. Ther..

[213] Kim J.-H., Jo C.H., Kim H.-R., Hwang Y.-i. (2018). Comparison of immunological characteristics of mesenchymal stem cells from the periodontal ligament, umbilical cord, and adipose tissue. Stem Cells Int..

[214] Subbarayan R., Murugan Girija D., Mukherjee J., Mamidanna S.R.R., Ranga Rao S. (2017). Comparision of gingival and umbilical cord stem cells based on its modulus and neuronal differentiation. J. Cell. Biochem..

[215] Wang Q., Zhao G., Xing Z., Zhan J., Ma J. (2019). Comparative evaluation of the osteogenic capacity of human mesenchymal stem cells from bone marrow and umbilical cord tissue. Exp. Ther. Med..

[216] Cabrera-Perez R., Monguio-Tortajada M., Gamez-Valero A., Rojas-Marquez R., Borras F.E., Roura S., Vives J. (2019). Osteogenic commitment of Wharton’s jelly mesenchymal stromal cells: Mechanisms and implications for bioprocess development and clinical application. Stem Cell Res. Ther..

[217] Das M., Das A., Barui A., Paul R.R. (2021). Comparative evaluation of proliferative potential and replicative senescence associated changes in mesenchymal stem cells derived from dental pulp and umbilical cord. Cell Tissue Bank..

[218] Tanhehco Y.C., Bhatia M. (2019). Hematopoietic stem cell transplantation and cellular therapy in sickle cell disease: Where are we now?. Curr. Opin. Hematol..

[219] Xu L., Liu Z., Wu Y., Yang X., Cao Y., Li X., Yan B., Li S., Da W., Wu X. (2018). Clinical evaluation of haploidentical hematopoietic combined with human umbilical cord-derived mesenchymal stem cells in severe aplastic anemia. Eur. J. Med. Res..

[220] Li X.-Y., Sun X., Chen J., Qin M.-Q., Luan Z., Zhu Y.-P., Fang J.-P. (2018). Hematopoietic stem cell transplantation for children with β-thalassemia major: Multicenter experience in China. World J. Pediatr..

[221] Dolstra H., Roeven M.W., Spanholtz J., Hangalapura B.N., Tordoir M., Maas F., Leenders M., Bohme F., Kok N., Trilsbeek C. (2017). Successful transfer of umbilical cord blood CD34+ hematopoietic stem and progenitor-derived NK cells in older acute myeloid leukemia patients. Clin. Cancer Res..

[222] Kim S.W., Han H., Chae G.T., Lee S.H., Bo S., Yoon J.H., Lee Y.S., Lee K.S., Park H.K., Kang K.S. (2006). Successful stem cell therapy using umbilical cord blood-derived multipotent stem cells for Buerger’s disease and ischemic limb disease animal model. Stem Cells.

[223] Ichim T.E., Solano F., Brenes R., Glenn E., Chang J., Chan K., Riordan N.H. (2008). Placental mesenchymal and cord blood stem cell therapy for dilated cardiomyopathy. Reprod. Biomed. Online.

[224] Laskowitz D.T., Bennett E.R., Durham R.J., Volpi J.J., Wiese J.R., Frankel M., Shpall E., Wilson J.M., Troy J., Kurtzberg J. (2018). Allogeneic umbilical cord blood infusion for adults with ischemic stroke: Clinical outcomes from a phase I safety study. Stem Cells Transl. Med..

[225] Hong B., Lee S., Shin N., Ko Y., Kim D., Lee J., Lee W. (2018). Bone regeneration with umbilical cord blood mesenchymal stem cells in femoral defects of ovariectomized rats. Osteoporos. Sarcopenia.

[226] Zhang W., Wang Y., Kong J., Dong M., Duan H., Chen S. (2017). Therapeutic efficacy of neural stem cells originating from umbilical cord-derived mesenchymal stem cells in diabetic retinopathy. Sci. Rep..

[227] Mohamed E.M., Abdelrahman S.A., Hussein S., Shalaby S.M., Mosaad H., Awad A.M. (2017). Effect of human umbilical cord blood mesenchymal stem cells administered by intravenous or intravitreal routes on cryo-induced retinal injury. IUBMB life.

[228] He B., Li X., Yu H., Zhou Z. (2015). Therapeutic potential of umbilical cord blood cells for type 1 diabetes mellitus. J. Diabetes.

[229] Boroujeni Z.N., Aleyasin A. (2014). Human umbilical cord-derived mesenchymal stem cells can secrete insulin in vitro and in vivo. Biotechnol. Appl. Biochem..

[230] Sun X., Hao H., Han Q., Song X., Liu J., Dong L., Han W., Mu Y. (2017). Human umbilical cord-derived mesenchymal stem cells ameliorate insulin resistance by suppressing NLRP3 inflammasome-mediated inflammation in type 2 diabetes rats. Stem Cell Res. Ther..

[231] Boroujeni M.E., Gardaneh M. (2017). Umbilical cord: An unlimited source of cells differentiable towards dopaminergic neurons. Neural Regen. Res..

[232] Ebrahimi M.J., Aliaghaei A., Boroujeni M.E., Khodagholi F., Meftahi G., Abdollahifar M.A., Ahmadi H., Danyali S., Daftari M., Sadeghi Y. (2018). Human Umbilical Cord Matrix Stem Cells Reverse Oxidative Stress-Induced Cell Death and Ameliorate Motor Function and Striatal Atrophy in Rat Model of Huntington Disease. Neurotox. Res..

[233] Cui Y., Ma S., Zhang C., Cao W., Liu M., Li D., Lv P., Xing Q., Qu R., Yao N. (2017). Human umbilical cord mesenchymal stem cells transplantation improves cognitive function in Alzheimer’s disease mice by decreasing oxidative stress and promoting hippocampal neurogenesis. Behav. Brain Res..

[234] Galieva L.R., Mukhamedshina Y.O., Arkhipova S.S., Rizvanov A.A. (2017). Human Umbilical Cord Blood Cell Transplantation in Neuroregenerative Strategies. Front. Pharmacol..

[235] Chez M., Lepage C., Parise C., Dang-Chu A., Hankins A., Carroll M. (2018). Safety and Observations from a Placebo-Controlled, Crossover Study to Assess Use of Autologous Umbilical Cord Blood Stem Cells to Improve Symptoms in Children with Autism. Stem Cells Transl. Med..

[236] Abo-Elkheir W., Hamza F., Elmofty A.M., Emam A., Abdl-Moktader M., Elsherefy S., Gabr H. (2017). Role of cord blood and bone marrow mesenchymal stem cells in recent deep burn: A case-control prospective study. Am. J. Stem Cells.

[237] Hashemi S.S., Mohammadi A.A., Kabiri H., Hashempoor M.R., Mahmoodi M., Amini M., Mehrabani D. (2019). The healing effect of Wharton’s jelly stem cells seeded on biological scaffold in chronic skin ulcers: A randomized clinical trial. J. Cosmet. Dermatol..

[238] Yu S., Long J., Yu J., Du J., Ma P., Ma Y., Yang D., Fan Z. (2013). Analysis of differentiation potentials and gene expression profiles of mesenchymal stem cells derived from periodontal ligament and Wharton’s jelly of the umbilical cord. Cells Tissues Organs.

[239] George J.P., Chakravarty P., Chowdhary K.Y., Purushothama H., Rao J.A. (2015). Attachment and differentiation of human umbilical cord stem cells on to the tooth root surface with and without the use of fibroblast growth factor-an in vitro study. Int. J. Stem Cells.

[240] Li Y., Hou R., Wang Y., Lu B., Zhang J., Feng X., Liu Y., Cao Q. (2014). Fundamental study of application of umbilical cord mesenchymal stem cells to the periodontium to aid healing after autotransplantation of teeth. Br. J. Oral Maxillofac. Surg..

[241] Kadam S., Gautam S., Dwivedi A., Jain V. (2019). Treatment of gingival recession defect using human umbilical cord mesenchymal stem cells cultured on PCL based bone regenerating scaffold: A randomized controlled clinical study. Cytotherapy.

[242] Shang F., Liu S., Ming L., Tian R., Jin F., Ding Y., Zhang Y., Zhang H., Deng Z., Jin Y. (2017). Human umbilical cord MSCs as new cell sources for promoting periodontal regeneration in inflammatory periodontal defect. Theranostics.

[243] Sun X.-C., Wang H., Li J.-h., Zhang D., Yin L.-Q., Yan Y.-F., Ma X., Xia H.-F. (2020). Repair of alveolar cleft bone defects by bone collagen particles combined with human umbilical cord mesenchymal stem cells in rabbit. Biomed. Eng. Online.

[244] Chen Y., Yu Y., Chen L., Ye L., Cui J., Sun Q., Li K., Li Z., Liu L. (2015). Human umbilical cord mesenchymal stem cells: A new therapeutic option for tooth regeneration. Stem Cells Int..

[245] MacMillan M.L., Blazar B.R., DeFor T.E., Wagner J.E. (2009). Transplantation of ex-vivo culture-expanded parental haploidentical mesenchymal stem cells to promote engraftment in pediatric recipients of unrelated donor umbilical cord blood: Results of a phase I–II clinical trial. Bone Marrow Transplant..

[246] Rowland A.L., Miller D., Berglund A., Schnabel L.V., Levine G.J., Antczak D.F., Watts A.E. (2020). Cross-matching of allogeneic mesenchymal stromal cells eliminates recipient immune targeting. Stem Cells Transl. Med..

[247] Yang S., Huang S., Feng C., Fu X. (2012). Umbilical cord-derived mesenchymal stem cells: Strategies, challenges, and potential for cutaneous regeneration. Front. Med..

[248] Bongso A., Fong C.-Y. (2013). The therapeutic potential, challenges and future clinical directions of stem cells from the Wharton’s jelly of the human umbilical cord. Stem Cell Rev. Rep..

